# Analysis of the role of 13 major fimbrial subunits in colonisation of the chicken intestines by *Salmonella enterica *serovar Enteritidis reveals a role for a novel locus

**DOI:** 10.1186/1471-2180-8-228

**Published:** 2008-12-18

**Authors:** Debra J Clayton, Alison J Bowen, Scott D Hulme, Anthony M Buckley, Victoria L Deacon, Nicholas R Thomson, Paul A Barrow, Eirwen Morgan, Michael A Jones, Michael Watson, Mark P Stevens

**Affiliations:** 1 Division of Microbiology, Institute for Animal Health, Compton, Berkshire, RG20 7NN, UK; 2 Pathogen Genomics, Wellcome Trust Sanger Institute, Hinxton, Cambridge, CB10 1SA, UK; 3 School of Veterinary Medicine and Science, University of Nottingham, Sutton Bonington, Leicestershire, LE12 5RD, UK

## Abstract

**Background:**

*Salmonella enterica *is a facultative intracellular pathogen of worldwide importance. Over 2,500 serovars exist and infections in humans and animals may produce a spectrum of symptoms from enteritis to typhoid depending on serovar- and host-specific factors. *S*. Enteritidis is the most prevalent non-typhoidal serovar isolated from humans with acute diarrhoeal illness in many countries. Human infections are frequently associated with direct or indirect contact with contaminated poultry meat or eggs owing to the ability of the organism to persist in the avian intestinal and reproductive tract. The molecular mechanisms underlying colonisation of poultry by *S*. Enteritidis are ill-defined. Targeted and genome-wide mutagenesis of *S*. Typhimurium has revealed conserved and host-specific roles for selected fimbriae in intestinal colonisation of different hosts. Here we report the first systematic analysis of each chromosomally-encoded major fimbrial subunit of *S*. Enteritidis in intestinal colonisation of chickens.

**Results:**

The repertoire, organisation and sequence of the fimbrial operons within members of *S. enterica *were compared. No single fimbrial locus could be correlated with the differential virulence and host range of serovars by comparison of available genome sequences. Fimbrial operons were highly conserved among serovars in respect of gene number, order and sequence, with the exception of *safA*. Thirteen predicted major fimbrial subunit genes were separately inactivated by lambda Red recombinase-mediated linear recombination followed by P22/int transduction. The magnitude and duration of intestinal colonisation by mutant and parent strains was measured after oral inoculation of out-bred chickens. Whilst the majority of *S*. Enteritidis major fimbrial subunit genes played no significant role in colonisation of the avian intestines, mutations affecting *pegA *in two different *S*. Enteritidis strains produced statistically significant attenuation. Plasmid-mediated *trans*-complementation partially restored the colonisation phenotype.

**Conclusion:**

We describe the fimbrial gene repertoire of the predominant non-typhoidal *S. enterica *serovar affecting humans and the role played by each predicted major fimbrial subunit in intestinal colonisation of the primary reservoir. Our data support a role for PegA in the colonisation of poultry by *S*. Enteritidis and aid the design of improved vaccines.

## Background

Non-typhoidal serovars of *Salmonella enterica *are an important cause of food-borne diarrhoeal illness in humans worldwide. Using active surveillance data from a catchment area of 44.5 million people, the FoodNet network has estimated that there are 1.4 million cases of human non-typhoid salmonellosis in the United States per annum, leading to 15,000 hospitalisations and 400 deaths [1]. Over the past three decades *S. enterica *serovar Enteritidis has emerged as a significant cause of such infections [2]. The consumption of undercooked poultry meat and eggs is a major risk factor for *S*. Enteritidis infection [3] and the phage types circulating in humans are commonly found in broilers [4] and layers [5]. The incidence of *S*. Enteritidis infection in humans declined markedly following the implementation of control strategies, including vaccination for poultry, regulations on storage and preparation of food and improved education [6]. Despite such measures, *S*. Enteritidis remains the most prevalent cause of non-typhoidal salmonellosis in many countries, including the United Kingdom , and improved vaccines are needed to achieve further reductions in the burden of human disease.

It is well established that *S*. Enteritidis is able to persist in the intestinal and reproductive tract of poultry in the absence of clinical signs [7]; however the molecular mechanisms mediating colonisation of these sites are ill-defined. Further, it is unclear why some *S. enterica *serovars are associated with enteric disease in a broad range of healthy out-bred adult hosts (e.g. Enteritidis and Typhimurium), whereas others are host-restricted or -specific and associated with severe systemic disease (e.g. Gallinarum in poultry and Typhi in humans). Targeted and genome-wide mutagenesis of the broad host range serovar Typhimurium has indicated that it uses both conserved and host-specific factors to colonise the intestines of chickens, cattle, pigs and mice [8-14]. Among the factors that influence intestinal colonisation are fimbriae; proteinaceous surface appendages that mediate interactions between bacteria and host cells.

Of the thirteen fimbrial loci predicted to be encoded by the *S*. Typhimurium genome, *lpf, fim, bcf, stb, stc, std, sth *and *csg *have been implicated in virulence in mice [11,13,15-17]. Screening of a library of signature-tagged mutants of *S*. Typhimurium indicated that pathogenicity island (SPI)-6-encoded *saf *fimbriae may play a host-specific role in ileal colonisation of pigs [14], whereas the *stbC, csgD *and *sthB *fimbrial genes were implicated in colonisation of the avian gut [12]. Separately Ledeboer et al described a role for *lpfA-E*, *pefC*, *csgA *and *fimH*, but not *sthD *or *bcfF*, in biofilm formation on chicken intestinal mucosa cultured *ex vivo *[18]. Relatively few studies have probed the role of fimbriae in colonisation of poultry by *S*. Enteritidis. Allen-Vercoe and Woodward reported that a *S*. Enteritidis mutant lacking *fimD*, *csgA*, *pefC*, *lpfC *and *sefA *colonised the caeca at comparable levels to the parent strain following oral dosing of 1 or 5 day-old chicks [19] and was similarly invasive [20] and adherent to chicken gut explants [21]. Furthermore, single mutants lacking *fimA, csgA *or *sefA *exhibited no significant defect in colonisation of chick caeca and were excreted in the faeces at comparable levels to the parent [22,23]. Although roles for *S*. Enteritidis fimbriae in intestinal colonisation of poultry have so far been lacking, Type I fimbriae [24] and curli [25] have been implicated in egg contamination.

In the recent publication of the complete genome sequence of *S*. Enteritidis strain P125019 [26] we have defined the full repertoire of fimbrial loci and identified a unique fimbrial operon, *peg*, present in *S*. Gallinarum, *S*. Enteritidis and also *S*. Paratyphi. The *peg *operon displays 60–70% sequence conservation with the *stc *operon of *S*. Typhimurium and is located in the same relative position. The *peg *operon belongs to the γ clade of fimbriae and is predicted to be assembled via the chaperone usher pathway [27].

The work herein examined the fimbrial gene conservation in the published genomes of other *S. enterica *serovars and also searched for traits associated with phase variation. Isogenic *S*. Enteritidis mutants with insertions in the major fimbrial subunit of each of the fimbrial operons were constructed using lambda Red recombinase-mediated linear recombination [28] followed by P22/int transduction. Mutant phenotypes were then evaluated and confirmed using an established chicken colonisation model.

## Methods

### *In silico *analysis of fimbrial loci

The complete genome sequences of *S. enterica *serovar Enteritidis strain P125109 [26], *S*. Gallinarum strain 287/91 [26], *S*. Typhimurium SL1344 and *S*. Typhimurium DT104 were produced by the Pathogen Sequencing Unit, Wellcome Trust Sanger Institute, UK . Published genome sequences were obtained from the National Center for Biotechnology Information (NCBI) and are described with their RefSeq-curated accession numbers; *S*. Typhimurium LT2 NC_003197 [29], *S*. Typhi CT18 NC_003198 [30], *S*. Typhi Ty2 NC_004631 [31] and *S*. Choleraesuis SC-B67 NC_006905 [32]. Fimbrial gene sequences were identified from the primary literature and databases via NCBI Entrez and the genome sequences were visualised and compared using Artemis and Artemis Comparison Tool ACT [33,34]. Direct and indirect repeat sequences were searched for as described [35]. A Perl script was written to isolate and visualise *S*. Enteritidis fimbrial operons and is available from the authors on request.

### Bacterial strains and plasmids

*S*. Enteritidis phage type 4 strain P125109 (NCTC 13349) was isolated from a poultry-associated outbreak in the UK and is naturally nalidixic acid resistant. A spontaneous nalidixic acid resistant derivative of *S*. Enteritidis S1400 [19] was selected by standard methods and it exhibits wild-type growth and chick colonisation phenotypes (data not shown). Strains were cultured in Luria-Bertani (LB) medium supplemented with antibiotics at the following concentrations where appropriate: nalidixic acid (Nal, 20 μg ml^-1^), novobiocin (1 μg ml^-1^), ampicillin (100 μg ml^-1^) and chloramphenicol (25 μg ml^-1^). Plasmids pCP20 [36], pKD3 and pKD46 [28] were obtained from the *E. coli *Genetic Stock Centre, Yale University. Plasmids pCR4Blunt-TOPO (Invitrogen, Paisley, UK) and pACYC177 [37] were used for cloning in *E. coli *K-12 strain TOP10F' (Invitrogen, Paisley, UK).

### Construction and validation of major fimbrial subunit mutations

Primers were designed to amplify the pKD3-encoded chloramphenicol resistance cassette, including 40 bp homology extensions from the 5' and 3' of each predicted major fimbrial subunit gene (Table 1). The extensions were designed such that the region between the start and stop codon of each major fimbrial subunit gene would be replaced by the chloramphenicol resistance cassette. PCR products were purified and electroporated into *S*. Enteritidis harbouring the helper plasmid pKD46, following induction of the Red recombinase with 10 mM L-arabinose at 30°C as previously described [28]. Recombinants were selected on LB-agar containing chloramphenicol and cured of pKD46 by culture at 37°C in the absence of ampicillin. Mutations were confirmed at the expected position in the genome by PCR with primers specific to the chloramphenicol resistance cassette and primers flanking each major fimbrial subunit gene (Table 2). Mutations were also confirmed by Southern blotting with *Hind*III-digested genomic DNA from wild-type and mutant strains using the *cat *gene as a probe. Attempts were made to transduce each mutation using bacteriophage P22/int into an archived strain to reduce the likelihood that phenotypes are the result of second site defects. For unknown reasons, three mutations could not be transduced, therefore the original recombinant was compared relative to the parent strain. Growth kinetics of all mutants were determined by diluting an overnight culture of *S*. Enteritidis wild-type or mutant strain 1:1000 in LB medium and measuring the absorbance at 600 nm every 30 minutes for 24 hours using a Bioscreen-C real-time spectrophotometer (Thermo^®^, Helsinki, Finland).

**Table 1:** Primers used to construct major fimbrial subunit mutations in *S*. Enteritidis P125109

**Name**	**Sequence (5'-3')**
stbAFmut	ATGTCTATGAAAAAATATTTAGCAATGATCACAGGCTCGCTGTGTAGGCTGGAGCTGCTTCG

stbARmut	TTATTTATACGAAACGGCGTATTGTAGGGTGGCAGCGACTCATATGAATATCCTCCTTA

pegAFmut	ATGAAACGTTCACTTATTGCTGCTTCTGTATTGTCTGCTGTGTGTAGGCTGGAGCTGCTTCG

pegARmut	TTAATCAGTTAATACCGTCATCGTCAGTACAGATTCAACACATATGAATATCCTCCTTA

stdAFmut	GTGCTTCGTTTAACACCAGGCGTTTATTATTCATACGAATTGTGTAGGCTGGAGCTGCTTCG

stdARmut	TCACAGGTATTTCAGGGTGTAGGTGACGGATGCGTTGAAGCATATGAATATCCTCCTTA

steAFmut	ATGAAGTCATCTCATTTTTGTAAACTGGCAGTAACTGCATGTGTAGGCTGGAGCTGCTTCG

steARmut	TTACAGGTAAGAGATAGTGACGTTGGCGGCGCTGCTGAACATATGAATATCCTCCTTA

stfAFmut	ATGAATACAGCAGTAAAAGCTGCGGTTGCTGCCGCACTGGTGTGTAGGCTGGAGCTGCTTCG

stfARmut	TTACAGATAGCTGATCGTGAAGTTTACGGTGCTGCTGAATCATATGAATATCCTCCTTA

sthAFmut	ATGTTTAATAAGAAAATTATCATCCTGGCAATGTTAACTTGTGTAGGCTGGAGCTGCTTCG

sthARmut	TTACTGATACGAAACGGTATACGTAACCTGAGTGCTAACACATATGAATATCCTCCTTA

stiAFmut	ATGAAACTCTCCTTAAAAACACTCACTGTGGCACTGCCGTGTGTAGGCTGGAGCTGCTTCG

stiARmut	TCAGTTATATTGCAGATAGAATGTTGCGGTTGCATCGACCCATATGAATATCCTCCTTA

bcfAFmut	ATGAAAAAGCCTGTACTAGCATTAATGGTCTCTGCCATTGTGTGTAGGCTGGAGCTGCTTC

bcfARmut	TCAGGAATAAACCATGCTAAATGTCGCCGTCGCGGTAACCATATGAATATCCTCCTTA

csgAFmut	ATGAAACTTTTAAAAGTGGCAGCATTCGCAGCAATCGTAGTTGTGTAGGCTGGAGCTGCTTCG

csgARmut	TTAATACTGGTTAGCCGTGGCGTTGTTGCCAAAACCAACCCATATGAATATCCTCCTTA

lpfAFmut	ATGGAGTTTTTAATGAAAAAGGTTGTTTTTGCTCTGTCTGTGTGTAGGCTGGAGCTGCTTCG

lpfARmut	TTATTCGTAGGACAGGTTGAAGTCACTTCTGCGTTACCGCATATGAATATCCTCCTTA

fimAFmut	ACCTCTACTATTGCGAGTCTGATGTTTGTCGCTGGCGCATGTGTAGGCTGGAGCTGCTTCG

fimARmut	TTATTCGTATTTCATGATAAAGGTGGCGTCGGCATTAGCCTGCATATGAATATCCTCCTTA

sefAFmut	ATGCGTAAATCAGCATCTGCAGTAGCAGTTCTTGCTTTAATGTGTAGGCTGGAGCTGCTTCG

sefARmut	GTTTTGATACTGCTGAACGTAGAAGGTCGCAGTGGGTCCATTTCATATGAATATCCTCCTTA

safAFmut	GTGGTTATTCAAATGAAAAGCATAAAAAAATTGATTATCGTGTGTAGGCTGGAGCTGCTTCG

safARmut	TTAAGGCTGATATCCCACTACGTCTACAGTTATTGGGTACCATATGAATATCCTCCTTA

The primers were designed to mutate the major fimbrial subunit by lambda Red recombinase-mediated integration of linear PCR products. Forward and reverse primers were used to amplify the pKD3-derived chloramphenicol cassette and contain 40 bp homology extensions 5' and 3' of the target gene.

**Table 2:** Primer combinations used to validate each fimbrial mutation.

**Primer combination**	**Predicted amplicon size (bp)**	**Sequence (5'-3')**
bcfAFOR + C1	633	TGCACTATCCGCAACGATATATTT

bcfAREV + C2	507	TAAAATACGCTTTCGCGATCGGTCGGT

csgAFOR + C2	173	CAAGGAGCAATAAAGTATGCATAATTT

csgAREV + C1	302	CAGCAGTTGTAGTGCAGAAACAGTCGCATA

lpfAFOR + C2	867	TTAGTTACGCGCTGTGTCAA

lpfAREV + C1	288	ATCCAATACCCACCTCTATACACTCCA

fimAFOR +C1	807	AACCTCAGATCGCACCTGCTGC

fimAREV + C2	429	ATGCCGACATGACGCCAGACC

sefAFOR + C1	373	CTATTAATGGGGATGTTGTGTAA

sefAREV + C2	946	CTAATAATCTCTTATAATTTC

safAFOR + C1	701	TGAGACTCTCTCATTGGAGCGCT

safAREV + C2	597	AATTGAGGTCAAGGGTCGCGCC

stbAFOR + C2	887	TTAATGGTGGGGGACATCGTA

stbAREV + C1	295	TTATTTTTACCACTCCATAAGCACGAA

pegAFOR + C2	179	CACAAGCCAGGCATAATGCAATCATC

pegAREV + C1	377	ACATTGCGATAACTTCCTGTCTATGAGAA

stdAFOR + C2	587	GCTGTACCGTACCTGACTGTC

stdAREV + C1	714	TGTTTTTAAATTTCATCCGCGAAG

steAFOR + C1	739	TACGACAACGCCTATATAATA

steAREV + C2	600	AGCAGCGTGGAGTGTCCCAGGTCAGC

stfAFOR + C1	283	CATATAAACATGGGGTATTGATGA

stfAREV + C2	155	GGCTGGCATCATCTTTAACA

sthAFOR + C1	584	GCGTTGATTTTGTTAATGC

sthAREV + C2	704	GAAAGCTCACGATTTGAGATCAAC

stiAFOR + C2	385	TTTGGCCGACAACACACTATG

stiAREV + C1	661	GTAAATCAGCTTAAATTCCG

C1	-	TTATACGCAAGGCGACAAGG

C2	-	GATCTTCCGTCACAGGTAGG

Primers are specific to the flanking regions of the specified fimbrial gene (FOR or REV) or the chloramphenicol resistance cassette (C1 – forward or C2 – reverse).

### FLP recombinase-mediated excision of the chloramphenicol resistance cassette

To remove the chloramphenicol resistance cassette from the Δ*pegA*::*cat *mutant and create a predicted non-polar mutation, the temperature-sensitive plasmid pCP20 was introduced and expression of FLP recombinase induced by culture at 42°C in the absence of antibiotic selection as described [28]. FLP-mediated recombination between flippase recognition target (FRT) sites flanking the pKD3-derived chloramphenicol resistance cassette was confirmed by PCR using primers flanking *pegA*. Excision of the chloramphenicol cassette was predicted to result in an 84 nucleotide in-frame scar between the *pegA *start and stop codons. The second codon in the scar is a stop codon, however *pegA *is not predicted to be translationally coupled to the 3' gene and therefore polar effects are not anticipated at the level of transcription or translation.

### *Trans*-complementation of the Δ*pegA::cat *mutant

The *pegA *coding sequence was amplified by PCR from *S*. Enteritidis P125109 genomic DNA using *Pfu *proof-reading DNA polymerase (Promega, Madison, USA) with primers *pegA-*for and *pegA*-rev containing *Cla*I restriction endonuclease cleavage sites. The *pegA *amplicon was ligated into pCR4Blunt-TOPO via topoisomerase I and transformed into chemically-competent *E. coli *TOP10 F' cells as described by the manufacturer (Invitrogen, Paisley, UK). A recombinant was verified by PCR with *pegA*-specific primers and digestion with *Cla*I. The *Cla*I fragment containing *pegA *was then sub-cloned into pACYC177 using T4 DNA ligase. Recombinant plasmids with the insert in the sense (p*pegA*_fwd_) and antisense (p*pegA*_rev_) orientation relative to the kanamycin resistance gene promoter of pACYC177 were isolated and electroporated into *S*. Enteritidis P125109 Δ*pegA*::*cat *with selection for ampicillin resistance.

### Experimental animals

Inoculation of chickens with *S*. Enteritidis wild-type, mutant and *trans*-complemented strains was conducted according to the requirements of the Animal (Scientific Procedures) Act 1986 (PPL 30/1998) with the approval of the local Ethical Review Committee. Specific pathogen-free out-bred Rhode Island Red chickens were reared at the Institute for Animal Health and housed in group cages in bio-secure accommodation. Birds were fed a vegetable-based diet (Special Diet Services, Manea, Cambridgeshire, UK) with access to water *ad libitum*. To reduce inter-animal variation, chickens were orally dosed on the day of hatch with 0.1 ml *Salmonella*-free adult gut flora cultured as described [38]. Owing to constraints of space, the phenotype of each fimbrial mutant could not be simultaneously evaluated relative to the parent. Rather, 4 groups of 15 birds were accommodated per room with 3 groups each receiving a different fimbrial mutant strain and one group the corresponding parent strain. Approximately 1.5 × 10^8 ^colony-forming units (CFU) of stationary phase LB-grown *Salmonella *were given by oral gavage at 18-days-old. Inocula were confirmed to be comparable by retrospective plating of serial dilutions to selective media. Five birds from each group were sacrificed by cervical dislocation at 3, 7 and 10 days post-inoculation and the liver, spleen, caecal contents, caecal wall, ileal contents and ileal wall were recovered aseptically and diluted 1:10 in phosphate-buffered saline for homogenisation. A rotary blade was used to homogenise the samples and serial ten-fold dilutions were plated on brilliant green agar containing novobiocin and nalidixic acid. As each sample was diluted 1:10 for homogenisation and 20 μl of this was plated in triplicate, the theoretical limit of detection by direct plating is log_10 _2.2 CFU/g. For some samples bacterial counts were below the limit of detection by direct plating and therefore enrichment was used. The homogenized sample was incubated overnight at 37°C in a final concentration of 1 × selenite broth before being plated on brilliant green agar plates supplemented with nalidixic acid and novobiocin. This results in a qualitative rather than a quantitative count but was given an arbitrary figure of log_10 _1 CFU/g as the sample diluted 10^-1 ^must have contained at least one viable organism. Owing to the difficulty separating caecal contents from the mucosa, the total caecal load is presented as a measure of colonisation of this site. This represents the mean viable count of *S*. Enteritidis in caecal content and mucosa samples, including biological and technical replicates.

To confirm the role of *pegA *in intestinal colonisation of chickens by *S*. Enteritidis, P125109 wild-type, Δ*pegA*::*cat *mutant, Δ*pegA *mutant, Δ*pegA*::*cat *[p*pegA*_fwd_] and Δ*pegA*::*cat *[p*pegA*_rev_] were given by oral gavage to ten 18-day-old Rhode Island Red chickens as above. Post mortem examinations were performed at 1 and 3 days post-inoculation (*n *= 5 per time interval) and bacteria at enteric and systemic sites enumerated. Plasmid stability in the absence of antibiotic selection *in vivo *was evaluated by plating selected samples to media containing nalidixic acid with or without ampicillin.

### Statistical analysis

Counts of viable bacteria were log_10 _transformed and a generalised linear model was constructed using the least square means ± standard error of the mean (Statistical Analysis System version 9, SAS Institute, Cary, NC, USA). The significance of differences between test and control groups was determined by an F-test with data taken as repeated measurements. *P *values < 0.05 were considered significant.

## Results

### *In silico *analysis of *S*. Enteritidis P125109 fimbrial loci

Fourteen predicted fimbrial loci of the sequenced *S*. Enteritidis phage type 4 strain were identified [26]. Thirteen fimbrial loci are predicted to be encoded on the genome, whereas the P125109 *pef *operon is plasmid-encoded and highly similar to that of *S*. Typhimurium LT2 and *S*. Choleraesuis SC-B67. As *S*. Enteritidis *pef *was previously reported to play no significant role in colonisation of 1- and 5-day-old chicks [19], we elected to focus this study on chromosomally-encoded loci. Additional file 1 shows the predicted organisation of each fimbrial operon of strain P125109, together with %G+C content of the locus and the location and e-values of Pfam subunit, usher and chaperone domains.

The analysis of the Pfam domains failed to identify a major fimbrial subunit in *csg *and *saf*, consistent with the prediction that they give rise to atypical fimbriae. The *csg *operon is not predicted to encode proteins with usher or chaperone domains, consistent with assembly of Csg fimbriae via a nucleator-dependent pathway [39]. The *saf *operon consists of a chaperone and usher domain. The adhesive component is formed by the main structural subunit whose sequence has been shown here to be highly variable and it is not located at the tip as with other chaperone/usher assembled fimbriae [40]. The *saf *fimbriae are composed of flexible linear multi-subunit fibers connected by short fibers or linkers which allow flexibility in the final structure [40].

**Figure 1:**
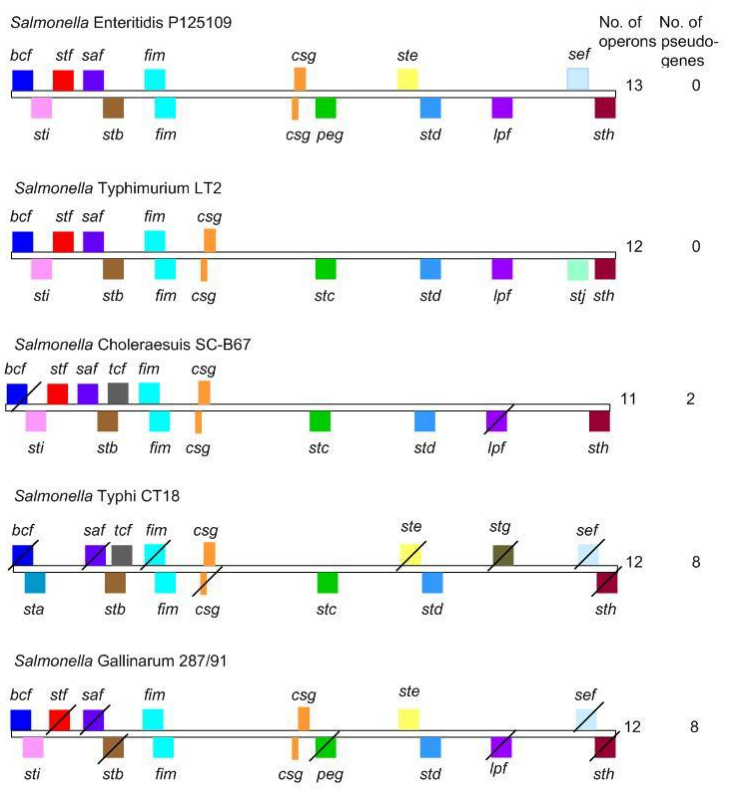
**Schematic representation of the repertoire and relative genomic location of fimbrial loci in the published genomes of *S. enterica *serovars**. Each coloured box represents a distinct fimbrial locus encoded in the sense (top) or anti-sense (bottom) orientation. Boxes of the same colour on both strands represent divergently transcribed operons. A diagonal line through the box indicates that at least one gene in the operon is a predicted pseudogene. The repertoire of *S*. Typhimurium and *S*. Typhi is representative of other sequenced strains of the same serovar. All genomes are aligned relative to their predicted origin. Not to scale.

The conservation and organisation of fimbrial loci in the genomes of sequenced strains of *S. enterica *was analysed using the Artemis Comparison Tool. This revealed differences in the number and location of fimbrial loci between the strains as well as the presence of predicted truncations and pseudogenes (Figure 1). At the nucleotide level, 56 of 71 fimbrial genes examined possessed ≥ 95% identity (Additional file 2). These include all of the genes of the fimbrial operons *sti, stb, fim, csg *and *lpf*, implying that their function may be conserved.

*S*. Enteritidis P125109 shares 10 of its 13 fimbriae with the sequenced *S*. Typhimurium strains. The *S*. Enteritidis *ste, sef *and *peg *operons are absent from the sequenced serovar Typhimurium strains, whereas the latter posses *stc *and *stj *operons that we do not find in P125109 (Figure 1).

We previously reported that the percentage of fimbrial genes that are pseudogenes in *S*. Gallinarum is greater than the genomic mean [26]. In addition we found here that the host-specific strains *S*. Typhi CT18 and *S*. Typhi Ty2 (data not shown) possessed the highest number of predicted fimbrial pseudogenes (based on the presence of at least one stop codon in the predicted coding sequence). The percentage of fimbrial genes that are pseudogenes in *S*. Typhi CT18 and *S*. Typhi Ty2 is 14% and 16% respectively, whereas the genomic mean of pseudogenes is 4.4%. In contrast the broad host-range serovars Enteritidis and Typhimurium LT2, DT104 and SL1344 appeared to contain an intact repertoire of fimbriae (data not shown) and the host-restricted serovar Choleraesuis maintained an intermediate number of predicted functional fimbrial genes. No single fimbrial locus could be correlated with host-specificity; however as has previously been suggested it is plausible that the loss of fimbrial genes in host-specific and -restricted serovars is associated with the narrowing of the niches they may occupy [26,29,30].

Fimbrial genes in some bacteria are subject to phase variable (on-off) expression that may be mediated via recombination (e.g. FimBE-mediated inversion of the *fimA *promoter in *E. coli *[41]), epigenetic regulation dependent on Dam methylation (e.g. control of Pap pili in uropathogenic *E. coli *[42]) or slipped-strand mis-pairing between homo- or hetero-polymeric tracts (e.g. assembly and maturation of Neisserial pilin reviewed in [43]). In *Salmonella*, evidence exists for phase variable expression of Type I fimbriae [44-46] and long-polar fimbriae [47]. Further, epigenetic regulation of the *pef *genes in *S*. Typhimurium by Dam methylation has been described [48] and expression of *std *fimbrial genes has been observed to be repressed in a *S*. Typhimurium Dam methylase mutant [49,50].

We searched *S*. Enteritidis fimbrial loci for traits associated with phase variation. Genes with homology to known recombinases were not detected within or proximal to fimbrial loci. Putative transposase and integrase genes associated with DNA mobility were observed proximal to the *saf*, *sef *and *fim *operons. Direct or inverted repeat sequences that may serve as substrates for recombination were not detected. A pattern-matching search was carried out for the Dam methylase target sequence GATC within and proximal to P125109 fimbrial operons. This identified hundreds of potential targets (Additional file 3), including those predicted to be methylated in the *S*. Typhimurium *pef *cluster [48]. Where *S*. Typhimurium strains SL1344 and LT2 possess GATC sites at -98, -110 and -212 relative to the start of *pefB*, *S*. Enteritidis P125109 possessed only the sites at -110 and -212, but an additional site at +47 in *pefB *that is absent in the two Typhimurium strains (Additional file 3a, grey shaded area). Three potential Dam methylation target sites were also identified upstream of the *std *operon (-88, -97 and -110) in *S*. Enteritidis P125109. This density of GATC sites is higher than random distribution would predict and correlates with the Dam-dependent repression of the *std *genes as detected by microarray analysis [49] and using antibody against StdA [50] (Additional file 3a, purple shaded area). Predicted Dam methylase targets were also identified upstream of the *sef*, *sti *and *stf *operons in *S*. Enteritidis P125109 (Additional file 3). Hundreds of homopolymeric tracts comprising 4 or more A or C residues were identified within fimbrial loci. Several conserved hetero-polymeric tracts were identified using a variable tandem repeat pattern finder, however only one was present in a fimbrial gene (ten repeated 6-mers (GACCAT) within *stdA*).

### Construction of *S*. Enteritidis major fimbrial subunit mutants

Amplicons for the 13 chromosomally-encoded predicted major fimbrial subunit genes of *S*. Enteritidis P125109, were produced in order to delete each one via lambda Red recombinase-mediated linear recombination. Despite repeated attempts, pKD46 failed to mediate homologous recombination of linear amplicons in *S*. Enteritidis P125109 under conditions suitable for other *S. enterica *strains. However all 13 genes were successfully disrupted in the *S*. Enteritidis phage type 4 strain S1400nal^R^, which is known to efficiently colonise the avian intestines [19,20]. Ten of the major fimbrial subunit gene deletions (marked by insertion of a chloramphenicol resistance cassette between the predicted start and stop codons) were successfully transduced into *S*. Enteritidis P125109 using bacteriophage P22/int. Transductants of *S*. Enteritidis P125109 or the archived S1400nal^R ^strain were not isolated for three of the mutated fimbrial constructs (Δ*safA*::*cat*, Δ*fimA*::*cat *and Δ*steA*::*cat)*. All of the successfully recovered isogenic mutants were verified by PCR and no growth defects were detected in batch culture (data not shown).

### Screening of *S*. Enteritidis fimbrial subunit mutants in a chick colonisation model

Although P125109 is known to colonise the intestines of streptomycin pre-treated mice [51], no data existed on the colonisation dynamics of the sequenced *S*. Enteritidis strain P125109 in chickens. A pilot experiment was therefore performed to evaluate the magnitude and duration of colonisation of enteric and systemic sites at intervals post-oral inoculation and to gain an assessment of inter-animal variation. Following oral gavage of 18-day-old out-bred specific pathogen-free Rhode Island Red chickens birds with 1.5 × 10^8 ^CFU, the caecal contents and mucosa were colonised by 4–5 log_10 _CFU/g of strain P125109 at days 1, 3 and 7 post-infection (*n *= 5 per time interval; Additional file 4). Bacterial colonisation of the ileum and translocation to the liver and spleen was detected, with recoveries at around the limit of detection at 2–3 log_10 _CFU/g by days 3 and 7 (Additional file 4).

**Figure 2:**
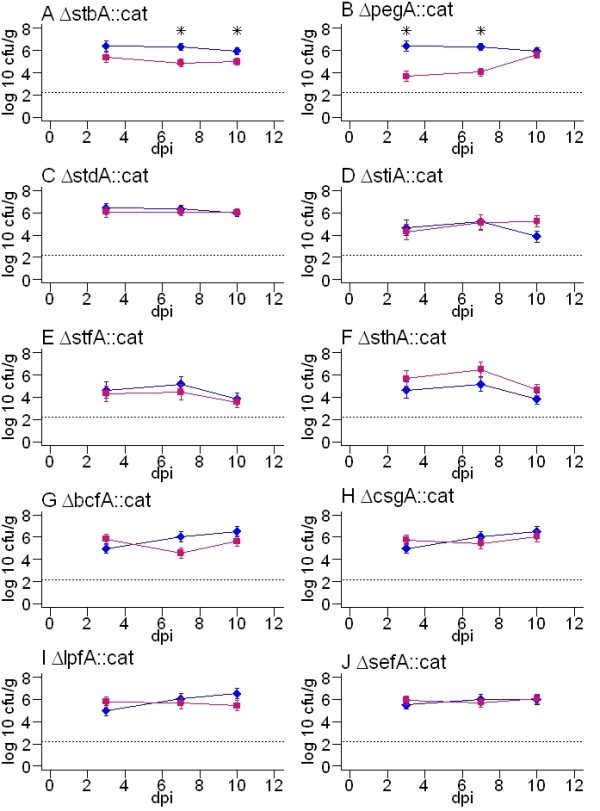
**Total caecal load of *S*. Enteritidis P125109 wild-type and major fimbrial subunit mutant strains at 3, 7 and 10 days post-oral inoculation of 18-day-old out-bred Rhode Island Red chickens**. Blue lines with diamonds denote the wild-type strain and pink lines with squares denote the fimbrial mutants. The dashed line indicates the theoretical limit of detection by direct plating (2.2 log_10 _CFU/g). The data reflect the mean ± standard error of the mean from five birds at each time interval. F-tests of the difference in recovery of wild-type and mutant strains at each time interval were performed and *P *values < 0.05 are marked with an asterisk.

**Figure 3:**
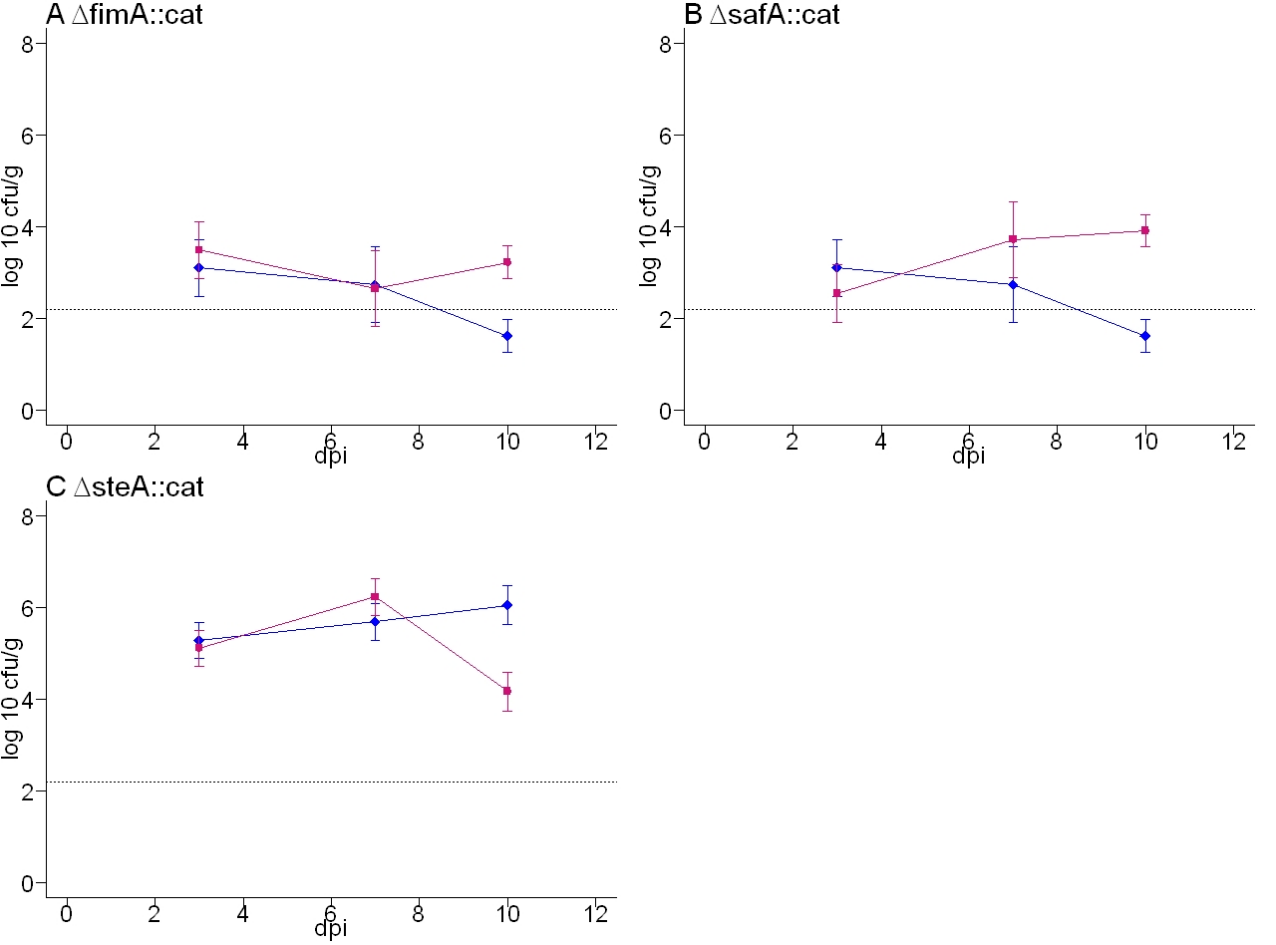
**Total caecal load of *S*. Enteritidis S1400nal^R ^wild-type, Δ*fimA*::*cat*, Δ*steA*::*cat *and Δ*safA*::*cat *mutant strains at 3, 7 and 10 days post-oral inoculation of 18-day-old out-bred Rhode Island Red chickens**. Blue lines with diamonds denote the wild-type strain and pink lines with squares denote the fimbrial mutants. The dashed line indicates the theoretical limit of detection by direct plating (2.2 log_10 _CFU/g). Samples positive only by enrichment culture were given an arbitrary value of 1 log_10 _CFU/g since at least one viable organism must have been present. The data reflect the mean ± standard error of the mean from five birds at each time interval. F-tests of the difference in recovery of wild-type and mutant strains at each time interval were performed and *P *values < 0.05 are marked with an asterisk.

Each fimbrial mutant was separately inoculated into groups of 15 Rhode Island Red chickens at 18 days-of-age and bacteria enumerated at enteric and systemic sites at 3, 7 and 10 days-post inoculation relative to the corresponding parent strain. As the caeca are a key site of bacterial persistence in the avian gut [52,53], (Additional file 4) and attenuation of defined and random *Salmonella *mutants is reliably detected at this site [12], the total caecal load is presented here as a measure of intestinal colonisation, representing the mean of the caecal wall and mucosa bacterial counts. We cannot preclude the possibility that some fimbriae mediate a specific tissue tropism that was not detected herein. Recoveries of viable bacteria from the liver and spleen were often close to the limit of detection by direct plating in chickens infected with wild-type strains (Additional file 4) and mutant strains (data not shown). Where adequate bacteria were recovered to permit a statistical analysis, no significant differences were observed at these sites. Figure 2 shows the caecal colonisation kinetics of *stb*,*peg, std*, *stf*, *sth*, *sti*, *bcf*, *csg*, *lpf *and *sef *major fimbrial subunit mutants of *S*. Enteritidis P125109 relative to the parent strain. The caecal loads of *fim*, *saf *and *ste *major fimbrial subunit mutants of *S*. Enteritidis strain S1400nal^R ^relative to the parent are shown in Figure 3.

The *S*. Enteritidis P125109 Δ*stbA*::*cat *fimbrial mutant was recovered from the chicken caeca at lower levels than the wild-type at all intervals post-inoculation (Figure 2A), with differences becoming significant by days 7 and 10 (*P *= 0.0081 and *P *= 0.03, respectively). This is consistent with the attenuation of a *S*. Typhimurium *stbC *mutant in chick caeca detected by signature-tagged mutagenesis [12]. The Δ*pegA*::*cat *mutant of *S*. Enteritidis P125109 was significantly impaired in colonisation of the caeca at days 3 and 7 post-inoculation compared with the wild-type (*P *= 0.0006 and *P *= 0.0002 respectively), although recoveries by day 10 were comparable (Figure 2B). The P125109 Δ*bcfA*::*cat *mutant, was recovered in significantly lower numbers than the parent strain at day 7 (*P *= 0.04), but not at other times (Figure 2G) and the S1400nal^R ^Δ*steA*::*cat *mutant was recovered in significantly lower numbers than the parent but only at day 10 (*P *= 0.0034; Figure 3C). No other fimbrial mutations significantly influenced the course of caecal colonisation at the 95% confidence interval.

### Confirmation of the role of PegA in colonisation of chickens by *S*. Enteritidis

**Figure 4:**
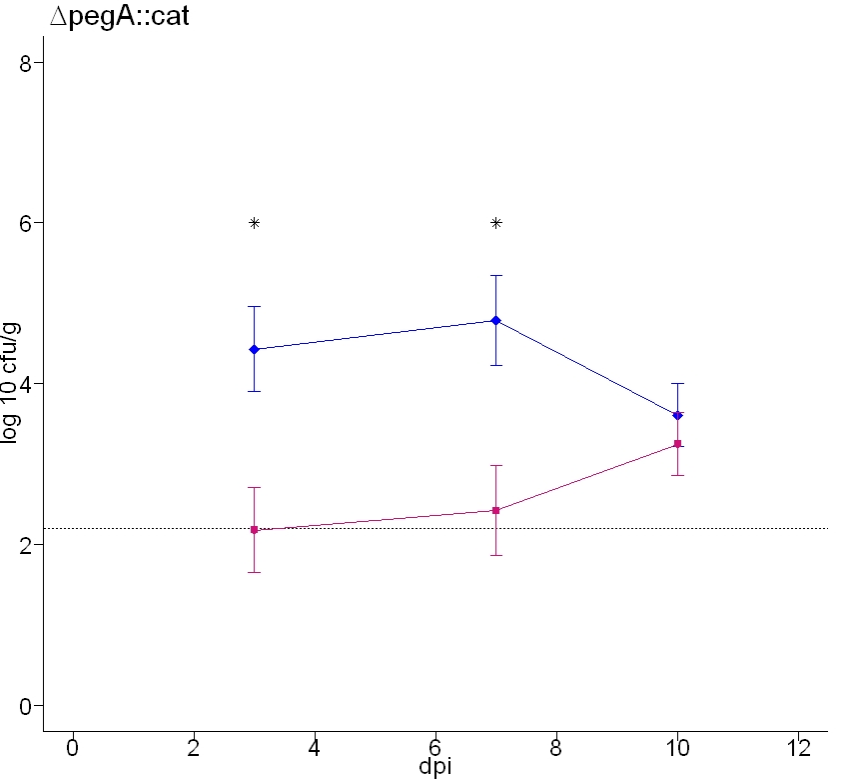
**Total caecal load of *S*. Enteritidis S1400nal^R ^wild-type and Δ*pegA*::*cat *fimbrial mutant strains at 3, 7 and 10 days post-oral inoculation of 18-day-old out-bred Rhode Island Red chickens**. Blue lines with diamonds denote the wild-type strain and pink lines with squares denote the fimbrial mutant. The dashed line indicates the theoretical limit of detection by direct plating (2.2 log_10 _CFU/g). The data reflect the mean ± standard error of the mean from five birds at each time interval. F-tests of the difference in recovery of wild-type and mutant strains at each time interval were performed and *P *values < 0.05 are marked with an asterisk.

Figure 2B implies a role for PegA in the colonisation of the chicken caeca. However, the Δ*pegA*::*cat *mutation was transduced from S1400nal^R ^into P125109 prior to analysis in chickens and a theoretical possibility exists that other traits proximal to the *pegA *gene were transferred that resulted in attenuation. To address this, we analysed the phenotype of the original S1400nal^R ^Δ*pegA*::*cat *mutant relative to the parent and sought to restore the mutant to the wild-type level of colonisation by plasmid-mediated *trans*-complementation using the same experimental design as above. As with the Δ*pegA*::*cat *mutant of P125109 (Figure 2B) an approximate 2 log_10 _CFU/g reduction in the total caecal load of the S1400nal^R ^Δ*pegA*::*cat *mutant was detected at days 3 and 7 post-inoculation relative to the parent strain (Figure 4; *P *= 0.0135 and *P *= 0.0088, respectively). However, as with the Δ*pegA*::*cat *mutant of strain P125109, no significant difference was detected by day 10 post-inoculation.

**Figure 5:**
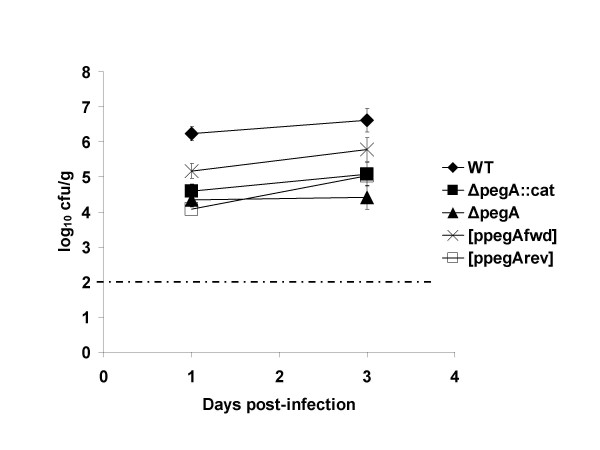
**Plasmid-mediated *trans-*complementation of the Δ*pegA*::*cat *mutant of *S*. Enteritidis P125109 at 1 and 3 days post-oral inoculation of 18-day-old out-bred Rhode Island Red chickens**. Total caecal load of the wild-type and mutant strain were compared to those of the P125109 Δ*pegA*::*cat *insertion mutant and Δ*pegA *strains in which *pegA *was introduced on plasmid pACYC177 in either the forward or reverse orientation relative to the promoter of the kanamycin resistance gene. The data represent the mean total caecal load ± standard error of the mean from five birds at each time interval for each strain. The dashed line indicates the theoretical limit of detection by direct plating (2.2 log_10 _CFU/g). F-tests of the difference in recovery of wild-type and mutant strains at each time interval were performed and *P *values < 0.05 are marked with an asterisk.

The chloramphenicol resistance cassette was excised from the P125109Δ*pegA*::*cat *fimbrial mutant to determine if polar effects on the expression of 3' genes may explain the attenuation observed. This addresses the possibility that *pegA *may not be involved in colonisation *per se*, but that downstream genes participate in the expression of surface structure(s) that may include distally-encoded fimbrial subunits. Excision was achieved by transient expression of flippase recombinase as described in the Methods. The total caecal loads of both the *S*. Enteritidis P125109 Δ*pegA*::*cat *andΔ*pegA *mutant were approximately two orders of magnitude lower than the parent strain at 1 and 3 days post-oral inoculation of chickens (Figure 5). No significant difference existed between the caecal loads of the Δ*pegA*::*cat *and Δ*pegA *mutants (*P *values 0.27 and 0.64 at 1 and 3 days post-inoculation, respectively); however in both cases a highly significant reduction in caecal load was detected for each mutant relative to the parent strain (*P *values < 0.0001 at 1 day post-infection), consistent with previous findings.

A pACYC177-derived plasmid was created in which the *S*. Enteritidis P125109 *pegA *gene was cloned in the same orientation as the kanamycin promoter (p*pegA*_fwd_), or in the antisense orientation (p*pegA*_rev_). This replicon was selected for *trans*-complementation as it did not impair the virulence of *S*. Typhimurium in mouse co-infection studies [54]. Introduction of p*pegA*_rev _into the *S*. Enteritidis P125109 Δ*pegA *mutant resulted in total caecal counts that were not significantly different to the Δ*pegA *fimbrial mutant at both 1 and 3 days post-oral inoculation of chickens (*P *= 0.24 and *P *= 0.07, respectively). However, recoveries of the p*pegA*_rev_-bearing strain were lower than for the Δ*pegA*::*cat *mutant alone at both time points, indicating that plasmid carriage may exert a slight fitness cost. The Δ*pegA *mutant harbouring p*pegA*_rev _was significantly attenuated compared to the parent strain at 1 and 3 days post-inoculation (*P *values < 0.0001). In contrast, introduction of the pACYC177-derived plasmid containing *pegA *in the sense orientation into the Δ*pegA *mutant partially restored the ability of the mutant strain to colonise the caeca at both time points relative to the wild-type strain (*P *= 0.0005 and *P *= 0.02 at 1 and 3 days post-inoculation, respectively) and to the Δ*pegA *fimbrial mutant (*P *= 0.0014 and *P *= 0.0005 at 1 and 3 days post-inoculation, respectively). Plating of tissue homogenates to media with or without ampicillin indicated that the plasmid was stably maintained in the absence of antibiotic selection *in vivo*. Taken together these data confirm that *pegA *plays a role in caecal colonisation of the avian intestines by *S*. Enteritidis.

## Discussion

*S*. Enteritidis phage type 4 is an important zoonotic pathogen and the factors mediating persistence in the avian reservoir are ill-defined. Toward an understanding of the molecular mechanisms by which *S*. Enteritidis colonises the chicken gut, the role of fimbriae was examined as these influence the carriage, virulence and tropism of other members of the Enterobacteriaceae. From the raw genome sequence of *S*. Enteritidis P125109, 13 intact chromosomal fimbrial loci were predicted. By comparing the sequence and distribution of the fimbrial loci with the published genomes of other *S. enterica *serovars *in silico*, no single locus correlated with host specificity. Microarray studies have indicated that a remarkable degree of conservation of fimbrial gene content exists among 26 *S*. Enteritidis isolates from varied geographical locations, hosts and years [55] and between strains of other broad host range serovars [56,57]. However, sequencing of such loci is required to determine if subtle differences in gene function exist.

Systematic mutagenesis of each major fimbrial subunit gene and screening in a chicken model revealed that the majority of major fimbrial subunits played no significant role in colonisation of the caeca (*P *values greater than 0.05). The absence of roles for *S*. Enteritidis Fim, Csg, Lpf and Sef fimbriae confirms previous reports that mutants lacking these fimbriae singly or in combination exhibit no significant defect in colonisation of chicks [19,20,22]. Conversely, the present study supports a role for Stb fimbriae in colonisation of the avian intestines by *Salmonella *that was suggested by the isolation of an *S*. Typhimurium *stbC *mutant by screening a library of signature-tagged mutants for attenuation in chicks [12]. The same screen of random mutants also identified attenuating mutations in *sthB *and *csgD*, however roles for *sthA *and *csgA *were not observed herein and studies with defined non-polar mutants and *trans*-complemented strains will be required to establish if the *sth *and *csg *loci play a conserved or serovar-specific role in colonisation of chickens. Owing to the relatively short-term nature of the studies reported here, we cannot preclude the possibility that the fimbrial subunits examined may play a role in longer-term persistence in the avian intestines or indeed tropism for the reproductive tract and egg, and further studies will be required to investigate this.

For the first time, we have shown that *S*. Enteritidis P125109 and S1400nal^R ^mutants of the novel Peg fimbrial operon show statistically significant attenuation in chickens that can be partially restored by plasmid mediated *trans*-complementation. A mutant in which the polar effects of the deletion of *pegA *are not predicted at the transcriptional or translational level was also attenuated; further implying that the phenotype of *pegA *insertion mutants is not due to altered expression of downstream genes. The inability of the p*pegA*_fwd _plasmid to fully restore colonisation to wild-type levels may reflect differences in the expression level of the fimbrial subunit *in vivo *and/or the fitness cost of maintaining the plasmid since recoveries of the Δ*pegA*::*cat *mutant bearing *pegA *on pACYC177 in the antisense orientation were slightly lower than for the mutant alone.

Assays with cultured cells did not indicate any significant role for *pegA *in adherence to primary chick kidney cells, HD11 avian macrophage-like cells or HEp-2 human laryngeal epithelial cells (data not shown) and there was no correlation between *in vitro *and *in vivo *results, regardless of the fimbriae examined. However, this is not unexpected as many fimbriae are known to be poorly expressed during culture in laboratory media [58], but are induced in bovine and murine intestinal lumen [58,59] and serve as antigens in mice [59].

Although there is attenuation of the *S*. Enteritidis *pegA *mutant, the *pegC *gene encoding a putative chaperone is a pseudogene in the sequenced strain of the poultry-adapted serovar *S*. Gallinarum, which implies that the possession of the entire fimbrial operon is unlikely to be a prerequisite for chicken colonisation. It should be noted that the tissue distribution of *S*. Enteritidis and *S*. Gallinarum is markedly different in age-matched healthy out-bred birds, with *S*. Gallinarum causing severe systemic disease with little enteric involvement whereas *S*. Enteritidis colonises the gut to a high level [7].

The absence of significant roles for *S*. Enteritidis fimbrial loci in isolation may reflect redundancy or the existence of compensatory mechanisms, whereby the loss of single fimbriae may modulate expression of other fimbriae or colonisation factors. In a murine model deletions in the *S*. Typhimurium *lpf*, *pef*, *fim *and *csg *operons only moderately impaired virulence when tested individually, whereas a mutant containing all four deletions exhibited a 26-fold increase in the median lethal dose and reduced ability to colonise the intestinal lumen [15]. Further studies with *S*. Enteritidis strains harbouring multiple fimbrial mutations may be warranted. Transcriptome analysis of the expression of fimbrial genes in the mutant strains described herein may indicate whether cross-talk and compensation mechanisms exist, provided probes are used that discriminate between fimbrial transcripts in the absence of cross-hybridisation.

## Conclusion

*S*. Enteritidis phage type 4 possesses thirteen chromosomally-encoded fimbrial loci, from which the predicted major fimbrial subunits of the majority can be deleted without significantly impairing caecal colonisation of chickens. Our data support the involvement of Stb fimbriae, previously suggested by screening of signature-tagged mutants of *S*. Typhimurium in poultry, and reveal for the first time that PegA influences caecal colonisation of chickens by *S*. Enteritidis. Since StbA and PegA serve as antigens in mice and vaccination with a cocktail of purified fimbrial subunits is partially protective in a murine model [59], further studies are required to evaluate the efficacy of subunit or live-attenuated vaccines that exploit the data presented here for control of zoonotic *S*. *enterica *serovars in poultry.

## Authors' contributions

DJC annotated and mutated the fimbrial loci, characterised each mutant *in vivo *and drafted the manuscript. AJB, SDH, AMB and VLD provided valuable assistance to the chicken colonisation studies. NRT and MW supported bioinformatic analysis of the fimbrial operons and co-supervised DJC. PAB originally conceived the study. MPS led study design, data interpretation and manuscript revisions. EM and MJ provided a supervisory role.

## Supplementary Material

Additional file 1 **Organisation of the fimbrial operons of *S*. Enteritidis P125109.** The image shows the gene organisation of each of the fimbrial operons, the Pfam domains within the fimbrial operons and the %GC content. Click here for file

Additional file 2 **Conservation of the nucleotide sequences of *S*. Enteritidis strain P125019 genes across sequenced strains of other *S. enterica *serovars.** The table provides the percent nucleotide identity of each fimbrial gene in several serovars of *Salmonella *compared with *S*. Enteritidis P125109. Click here for file

Additional file 3 **3a. Dam methylase target sequence GATC within and proximal to *S*. Enteritidis P125109 fimbrial operons.** 3b. Putative homo-polymeric tracts in the *S*. Enteritidis P125109 genome sequence. The tables indicate regions of potential phase variable targets. Click here for file

Additional file 4 ***S*****. Enteritidis P125109 colonisation of Rhode Island Red Chickens at 1, 3 and 7 days post-infection.** The graph shows the colonisation of *S*. Enteritidis P125109 at different organs within the chicken, at different times post-infection. Click here for file
