# Supplementary material for: Analysis of the role of 13 major fimbrial subunits in colonisation of the chicken intestines by Salmonella enterica serovar Enteritidis reveals a role for a novel locus
Source: BMC Microbiol. 2008 Dec 18;8:228. doi: 10.1186/1471-2180-8-228 (PMC2644700; doi:10.1186/1471-2180-8-228)

**Additional file 1**.

Organisation of the fimbrial operons of *S*. Enteritidis P125109. Arrows denote the direction of transcription. Blue arrows denote the location of predicted Pfam domains, red arrows show predicted co-ordinates of coding sequences and green arrows show miscellaneous features. The graph indicates the percentage GC content (to scale, different scale per operon). The Perl script used to generate the data is available from the authors on request.


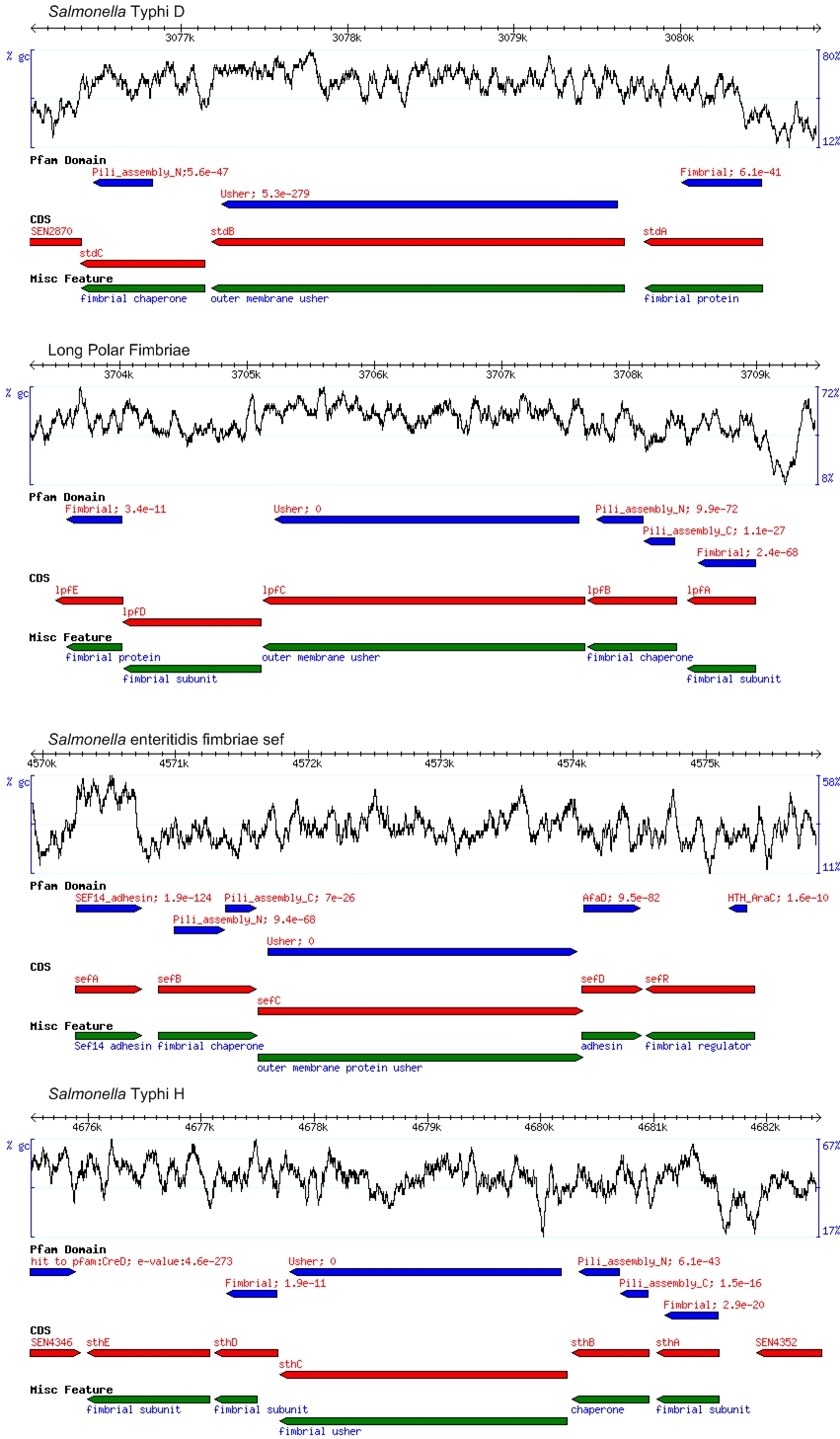

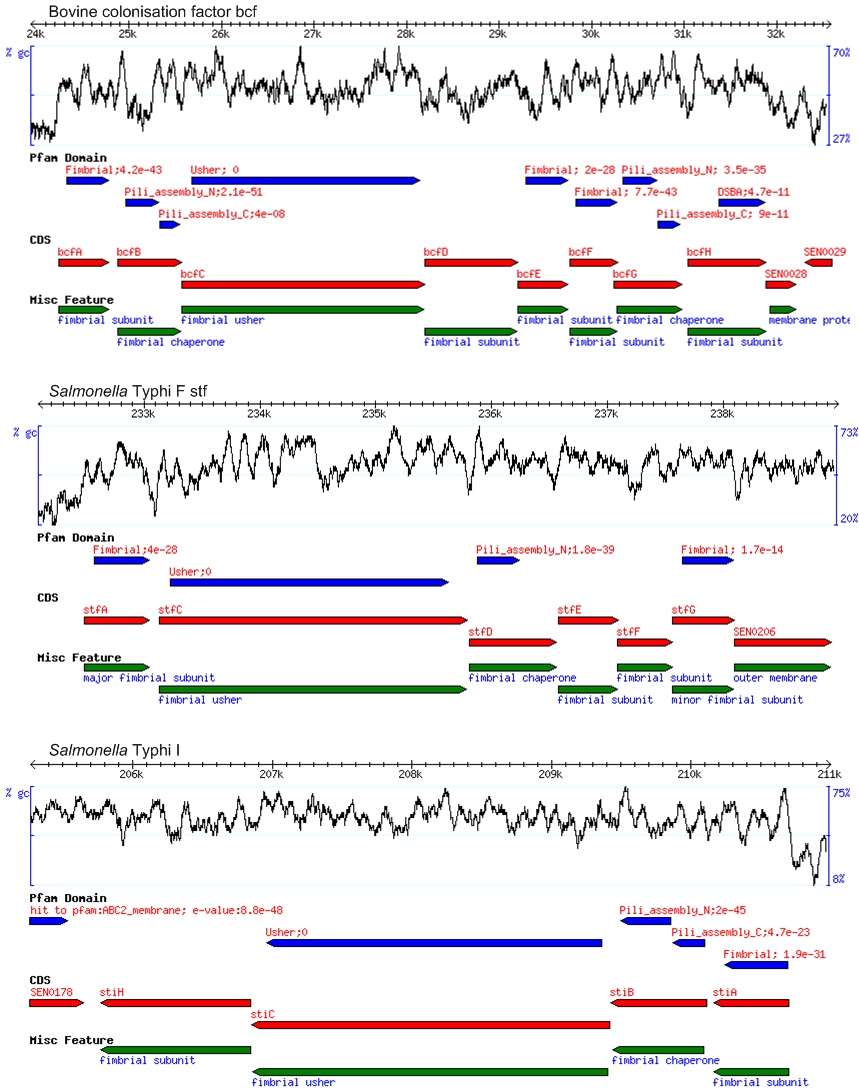

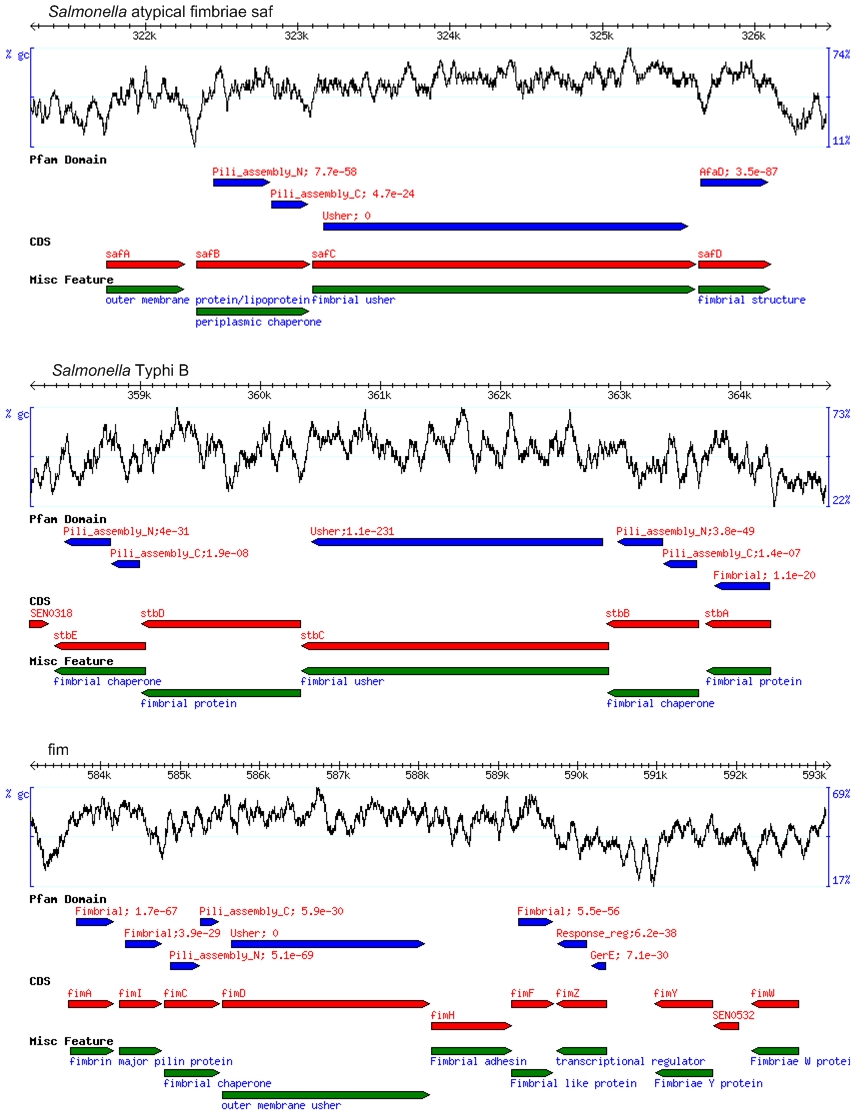

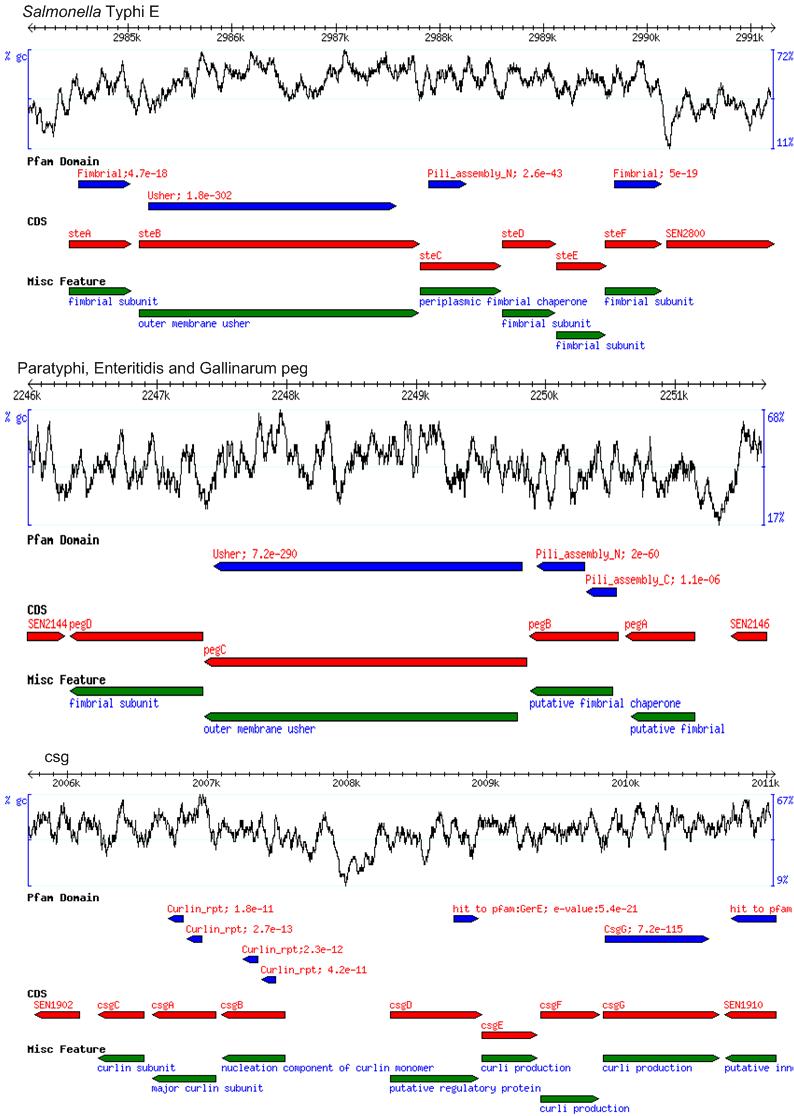

Supplement: Additional file 1 — Organisation of the fimbrial operons of S. Enteritidis P125109. The image shows the gene organisation of each of the fimbrial operons, the Pfam domains within the fimbrial operons and the %GC content. [file 1471-2180-8-228-S1.doc]
