# Supplementary material for: Analysis of the role of 13 major fimbrial subunits in colonisation of the chicken intestines by Salmonella enterica serovar Enteritidis reveals a role for a novel locus
Source: BMC Microbiol. 2008 Dec 18;8:228. doi: 10.1186/1471-2180-8-228 (PMC2644700; doi:10.1186/1471-2180-8-228)
Supplement: Additional file 2 — Conservation of the nucleotide sequences of S. Enteritidis strain P125019 genes across sequenced strains of other S. enterica serovars. The table provides the percent nucleotide identity of each fimbrial gene in several serovars of Salmonella compared with S. Enteritidis P125109. [file 1471-2180-8-228-S2.doc]

**Additional file 2**.

**Conservation of the nucleotide sequences of *S*. Enteritidis strain P125019 genes across sequenced strains of other *S. enterica* serovars.**

Percent identity to the *S*. Enteritidis P125109 gene was calculated using BLASTn. Abbreviations: n, length in nucleotides; X, gene absent; SE, *S.* Enteritidis P125109; SG, *S.* Gallinarum 287/91; STm, *S.* Typhimurium LT2; ST, *S.* Typhi CT18; SC, *S.* Choleraesuis SC-B67; P, pseudogene. The shading reflects the divergence of the *safA* gene and the *peg* or *stc* operons identified in the same chromosomal location but with limited homology.

|  | *SE* | *SG* | | *STm* | | *ST* | | *SC* | |
| --- | --- | --- | --- | --- | --- | --- | --- | --- | --- |
| Gene | n | n | % | n | % | n | % | n | % |
| *bcfA* | 543 | 543 | 99 | 543 | 99.63 | 543 | 98.89 | 543 | 99.63 |
| *bcfB* | 687 | 688 | 97.23 | 687 | 99.85 | 687 | 98.98 | 687 | 79.18 |
| *bcfC* | 2622 | 2622 | 99 | 2622 | 99.24 | 2622p | 98.36 | 2622p | 99.05 |
| *bcfD* | 1008 | 1008 | 100 | 1008 | 99.31 | 1008 | 98.12 | 1008 | 98.71 |
| *bcfE* | 546 | 546 | 100 | 546 | 98.35 | 546 | 97.62 | 546 | 98.90 |
| *bcfF* | 519 | 519 | 99 | 519 | 99.81 | 519 | 97.88 | 519 | 99.23 |
| *bcfG* | 732 | 732 | 100 | 732 | 99.32 | 705 | 95.63 | 732 | 99.45 |
| *bcfH* | 846 | 846 | 100 | 846 | 99.76 | 846 | 100 | 846 | 99.53 |
| *stiA* | 540 | 540 | 99 | 540 | 99.23 | X | X | 540 | 99.63 |
| *stiB* | 684 | 684 | 97 | 684 | 98.97 | X | X | 684 | 98.97 |
| *stiC* | 2547 | 2547p | 99.8 | 2547 | 99.72 | X | X | 2547 | 99.37 |
| *stiH* | 1080 | 1080 | 99 | 1080 | 99.35 | X | X | 1080 | 99.17 |
| *stfA* | 561 | 561 | 99 | 561 | 98.57 | X | X | 561 | 99.15 |
| *stfC* | 2658 | 2658 | 99 | 2658 | 99.59 | X | X | 2658 | 99.47 |
| *stfD* | 753 | 753 | 99 | 753 | 99.47 | X | X | 753 | 99.47 |
| *stfE* | 513 | 513 | 98 | 513 | 99.02 | X | X | 513 | 98.83 |
| *stfF* | 477 | 477p | 85 | 477 | 99.58 | X | X | 477 | 92.66 |
| *stfG* | 531 | 531 | 98 | 531 | 96.99 | X | X | 531 | 91.34 |
| *safA* | 510 | 495 | 51.2 | 513 | 81.48 | 495 | 68.63 | 513 | 81.18 |
| *safB* | 738 | 741 | 83.5 | 738 | 87.12 | 741 | 81.10 | 738 | 95.94 |
| *safC* | 2511 | 2482p | 50.8 | 2511 | 98.85 | 2511 | 98.49 | 2511 | 98.73 |
| *safD* | 471 | 471 | 93.6 | 471 | 96.39 | 471 | 96.60 | 471 | 95.54 |
| *stbA* | 537 | 537 | 100 | 537 | 98.88 | 537 | 98.69 | 537 | 99.26 |
| *stbB* | 762 | 762 | 99 | 762 | 99.21 | 762 | 98.03 | 762 | 98.95 |
| *stbC* | 2562 | 2559p | 99 | 2562 | 99.22 | 2562 | 98.83 | 2562 | 99.53 |
| *stbD* | 1326 | 1326 | 99 | 1326 | 99.02 | 1326 | 98.87 | 1326 | 99.25 |
| *stbE* | 759 | 756 | 99 | 759 | 97.76 | 759 | 98.95 | 760 | 98.82 |
| *fimA* | 558 | 556 | 98.6 | 558 | 98.75 | 555 | 97.83 | 555 | 99.95 |
| *fimI* | 534 | 534 | 100 | 534 | 98.32 | 534p | 99.06 | 534p | 98.50 |
| *fimC* | 693 | 693 | 98 | 693 | 98.99 | 693 | 98.70 | X | X |
| *fimD* | 2613 | 2613 | 99 | 2613 | 99.00 | 2613 | 98.81 | 2619 | 99.00 |
| *fimH* | 1008 | 1008 | 99 | 1008 | 98.41 | 1008 | 97.62 | 1008 | 98.81 |
| *fimF* | 519 | 519 | 100 | 519 | 99.23 | 519 | 98.07 | 519 | 99.81 |
| *fimZ* | 633 | 633 | 98 | 633 | 100 | 633 | 98.74 | 634 | 99.53 |
| *fimY* | 723 | 723 | 98 | 723 | 98.20 | 723 | 98.20 | 723 | 97.79 |
| *fimW* | 597 | 596 | 99 | 597 | 98.49 | 597 | 98.32 | 597 | 98.83 |
| *csgC* | 327 | 327 | 100 | 327 | 100 | 327 | 99.69 | 327 | 100 |
| *csgB* | 456 | 456 | 99 | 456 | 99.56 | 456 | 99.34 | 456 | 99.78 |
| *csgA* | 456 | 456 | 99 | 456 | 98.90 | 456 | 98.68 | 456 | 100 |
| *csgD* | 651 | 651 | 99 | 651 | 99.69 | 627 | 95.39 | 651 | 100 |
| *csgE* | 396 | 396 | 99 | 396 | 100 | 396 | 100 | 396 | 100 |
| *csgF* | 417 | 417 | 99 | 417 | 100 | 417 | 99.52 | 417 | 99.52 |
| *csgG* | 834 | 834 | 100 | 834 | 99.73 | 834 | 99.33 | 834 | 99.73 |
| *pegA/stcA* | 534 | 534 | 100 | 531 | 66.85 | 531 | 66.85 | 531 | 67.23 |
| *pegB/stcB* | 681 | 684 | 98 | 684 | 64.51 | 684 | 64.51 | 684 | 64.51 |
| *pegC/stcC* | 2487 | 2488p | 99 | 2490 | 66.25 | 2490 | 66.25 | 2490 | 66.25 |
| *pegD/stcD* | 1023 | 1023 | 98 | 1008 | 58.14 | 1008 | 58.14 | 1008 | 58.14 |
| *stdA* | 711 | X | X | 711 | 89.31 | 708 | 95.48 | 711 | 91.14 |
| *stdB* | 2484 | X | X | 2490 | 99.52 | 2490 | 97.63 | 2490 | 99.44 |
| *stdC* | 744 | X | X | 744 | 99.06 | 744 | 97.31 | 744 | 98.25 |
| *steA* | 588 | 588 | 100 | X | X | 588p | 98.13 | X | X |
| *steB* | 2700 | 2700 | 99 | X | X | 2700 | 99.23 | X | X |
| *steC* | 774 | 774 | 99 | X | X | 774 | 98.32 | X | X |
| *steD* | 507 | 504 | 99 | X | X | 507 | 99.21 | X | X |
| *steE* | 471 | 471 | 100 | X | X | 471 | 98.31 | X | X |
| *steF* | 537 | 537 | 100 | X | X | 537 | 85.66 | X | X |
| *lpfA* | 537 | 537 | 99 | 537 | 99.81 | X | X | 537 | 99.81 |
| *lpfB* | 699 | 699 | 100 | 699 | 99.86 | X | X | 699 | 100 |
| *lpfC* | 2529 | 2528p | 99 | 2529 | 99.20 | X | X | 2528p | 99.64 |
| *lpfD* | 1080 | 1080 | 99 | 1080 | 99.54 | X | X | 1080 | 99.54 |
| *lpfE* | 528 | 528 | 99 | 528 | 99.81 | X | X | 528 | 97.35 |
| *sefA* | 537 | 537 | 92.7 | X | X | 536p | 99.32 | X | X |
| *sefB* | 741 | 740 | 91.9 | X | X | 753 | 97.98 | X | X |
| *sefC* | 2445 | 2445p | 98 | X | X | 2517 | 99.79 | X | X |
| *sefD* | 453 | 452p | 100 | X | X | 443p | 97.79 | X | X |
| *sefR* | 837 | 837 | 53 | X | X | 812p | 95.10 | X | X |
| *sthA* | 546 | 546 | 99 | 546 | 98.35 | 546 | 98.71 | 546 | 98.72 |
| *sthB* | 684 | 684p | 99 | 684 | 99.71 | 684 | 97.91 | 684 | 98.90 |
| *sthC* | 2538 | 2454 | 93.1 | 2538 | 99.68 | 2534p | 98.70 | 2538 | 99.09 |
| *sthD* | 558 | 558 | 100 | 558 | 99.13 | 558 | 97.31 | 558 | 98.75 |
| *sthE* | 1086 | 1047 | 90 | 1086 | 99.36 | 1085p | 97.88 | 1086 | 98.98 |
