# Supplementary material for: Analysis of the role of 13 major fimbrial subunits in colonisation of the chicken intestines by Salmonella enterica serovar Enteritidis reveals a role for a novel locus
Source: BMC Microbiol. 2008 Dec 18;8:228. doi: 10.1186/1471-2180-8-228 (PMC2644700; doi:10.1186/1471-2180-8-228)
Supplement: Additional file 3 — 3a. Dam methylase target sequence GATC within and proximal to S. Enteritidis P125109 fimbrial operons. 3b. Putative homo-polymeric tracts in the S. Enteritidis P125109 genome sequence. The tables indicate regions of potential phase variable targets. [file 1471-2180-8-228-S3.doc]

**Additional file 3a**

**Dam methylase target sequence GATC within and proximal to *S*. Enteritidis P125109 fimbrial operons.**

The fimbrial gene downstream of the GATC site or the fimbrial gene in which the GATC is located is noted. The position of the GATC site relative to the gene is also included.

| **GATC genome location** | Relative location | **Fimbrial gene location** |
| --- | --- | --- |
| 24391-24394 | 156 bases into *bcfA* | *bcfA* 24235-24777 |
| 25564-25567 | Overlapping start of *bcfC* | *bcfC* 25569-28190 |
| 232155-232158 | 330 upstream of *stf* operon | *stfA* 232485-233045 |
| 232259-232262 | 226 upstream of *stf* operon | *stfA* 232485-233045 |
| 233275-233278 | 144 bases into *stfC* | *stfC* 233131-235788 |
| 235893-235896 | 87 bases into *stfD* | *stfD* 235806-236558 |
| 321303-321306 | 444 bases upstream of *safA* | *safA* 321747-322256 |
| 2007363-2007366 | 190 bases into *csgB* | *csgB* 2007101-2007556c |
| 2007481-2007484 | 75 bases into *csgB* | *csgB* 2007101-2007556c |
| 2008355-2008358 | 44 bases into *csgD* | *csgD* 2008311-2008961 |
| 2984272-2984275 | 163 bases upstream of *steA* | *steA* 2984435-2985022 |
| 2984588-2984591 | 153 bases into *steA* | *steA* 2984435-2985022 |
| 4569956-4569959 | 245 bases upstream of *sefA* | *sefA* 4570250-4570747 |
| 4570097-4570100 | 153 bases upstream of *sefA* | *sefA* 4570250-4570747 |
| 4570145-4570148 | 105 bases upstream of *sefA* | *sefA* 4570250-4570747 |
| 4570397-4570400 | 147 bases into *sefA* | *sefA* 4570250-4570747 |
| 583667-583670 | 69 bases into *fimA* | *fimA* 583598-584155 |
| 589175-589178 | 2 bases into *fimF* | *fimF* 589173-589691 |
| 2987904-2987907 | 90 bases into *steC* | *steC* 2987814-2988587 |
| 4677536-4677539 | 135 bases into *sthD* | *sthD* 4677117-4677674c |
| 4677600-4677603 | 74 bases into *sthD* | *sthD* 4677117-4677674c |
| 4680139-4680142 | 87 bases into *sthC* | *sthC* 4677692-4680229c |
| 4681823-4681826 | 249 bases upstream of *sthA* | *sthA* 4681029-4681574c |
| 4681472-4681475 | 99 bases into *sthA* | *sthA* 4681029-4681574c |
| 3080578-3080581 | 88 bases upstream of *stdA* | *stdA* 3079783-3080490c |
| 3080587-3080590 | 97 bases upstream of *stdA* | *stdA* 3079783-3080490c |
| 3080600-3080603 | 110 bases upstream of *stdA* | *stdA* 3079783-3080490c |
| 3080295-3080298 | 195 bases into *stdA* | *stdA* 3079783-3080490c |
| 364215-364218 | 29 bases into *stbA* | *stbA* 363708-364244c |
| 364336-364339 | 92 bases upstream of *stbA* | *stbA* 363708-364244c |
| 362854-362857 | 4 bases upstream of *stbC* | *stbC* 360340-362901c |
| 210707-210710 | 8 bases upstream of *stiA* | *stiA* 210160-210699c |
| 211099-211102 | 400 bases upstream of *stiA* | *stiA* 210160-210699c |
| 210991-210994 | 292 bases upstream of *stiA* | *stiA* 210160-210699c |
| 14610-14613 | 47 bases within *pefB* | *pefB* 14358-14657c |
| 14767-14770 | 110 bases upstream of *pefB* | *pefB* 14358-14657c |
| 14869-14872 | 212 bases upstream of *pefB* | *pefB* 14358-14657c |

**3b. Putative homo-polymeric tracts in the *S*. Enteritidis P125109 genome sequence**

Homo-polymeric repeats non-divisible by 3 at the beginning of a fimbrial gene were noted. Only A’s and C’s were searched for as obviously homo-polymeric tracts consisting of T’s or G’s will complement those identified from A’s and C’s. The location of the homo-polymeric tracts was noted along with the gene they were present in.

| **Repeats** | **Location** | **Gene** |
| --- | --- | --- |
| 5A | 24238-24242 | *bcfA* 24235-24777 |
| 4A | 24339-24342 | *bcfA* 24235-24777 |
| 8A | 24881-24888 | *bcfB* 24878-25564 |
| 4A | 24897-24900 | *bcfB* 24878-25564 |
| 7A | 25047-25053 | *bcfB* 24878-25564 |
| 4C | 25627-25630 | *bcfC* 25569-28190 |
| 4C | 25664-25667 | *bcfC* 25569-28190 |
| 4A | 25582-25585 | *bcfC* 25569-28190 |
| 4A | 25675-25678 | *bcfC* 25569-28190 |
| 4A | 25737-25740 | *bcfC* 25569-28190 |
| 4A | 28194-28197 | *bcfD* 28191-29198 |
| 4A | 28204-28207 | *bcfD* 28191-29198 |
| 5A | 28266-28270 | *bcfD* 28191-29198 |
| 4A | 28327-28330 | *bcfD* 28191-29198 |
| 6A | 28357-28362 | *bcfD* 28191-29198 |
| 5A | 29221-29225 | *bcfE* 29199-29744 |
| 4A | 29887-29890 | *bcfF* 29760-30278 |
| 4A | 29928-29931 | *bcfF* 29760-30278 |
| 4A | 29963-29966 | *bcfF* 29760-30278 |
| 4C | 30380-30383 | *bcfG* 30244-30975 |
| 5A | 30283-30287 | *bcfG* 30244-30975 |
| 4A | 30443-30446 | *bcfG* 30244-30975 |
| 4A | 31056-31059 | *bcfH* 31039-31884 |
| 5A | 31231-31235 | *bcfH* 31039-31884 |
| 4C | 31130-31133 | *bcfH* 31039-31884 |
| 4A | 232499-232502 | *stfA* 232485-233045 |
| 4A | 232580-232583 | *stfA* 232485-233045 |
| 4A | 232903-232906 | *stfA* 232485-233045 |
| 6A | 233143-233148 | *stfC* 233131-235788 |
| 4A | 233204-233207 | *stfC* 233131-235788 |
| 4A | 233287-233290 | *stfC* 233131-235788 |
| 4C | 233313-233317 | *stfC* 233131-235788 |
| 4C | 236087-236090 | *stfD* 235806-236558 |
| 5C | 236111-236115 | *stfD* 235806-236558 |
| 4A | 235817-235820 | *stfD* 235806-236558 |
| 4A | 235840-235843 | *stfD* 235806-236558 |
| 4A | 235849-235852 | *stfD* 235806-236558 |
| 5A | 235947-235951 | *stfD* 235806-236558 |
| 4C | 236766-236769 | *stfE* 236577-237089 |
| 5C | 237115-237119 | *stfF* 237086-237562 |
| 7A | 237089-237095 | *stfF* 237086-237562 |
| 4A | 237242-237245 | *stfF* 237086-237562 |
| 4A | 237568-237571 | *stfG* 237562-238092 |
| 5A | 237651-237655 | *stfG* 237562-238092 |
| 5A | 237720-237724 | *stfG* 237562-238092 |
| 4C | 237610-237613 | *stfG* 237562-238092 |
| 4A | 321762-321765 | *safA* 321747-322256 |
| 7A | 321770-321776 | *safA* 321747-322256 |
| 5A | 321831-321835 | *safA* 321747-322256 |
| 4A | 321886-321889 | *safA* 321747-322256 |
| 4A | 322343-322346 | *safB*  322340-323077 |
| 4A | 322354-322357 | *safB*  322340-323077 |
| 4A | 322359-322362 | *safB*  322340-323077 |
| 4A | 322536-322539 | *safB*  322340-323077 |
| 4C | 323209-323212 | *safC* 323101-325611 |
| 5C | 323235-323239 | *safC* 323101-325611 |
| 4A | 325642-325645 | *safD* 325633-326103 |
| 4A | 325657-325660 | *safD* 325633-326103 |
| 5A | 325727-325731 | *safD* 325633-326103 |
| 4C | 325753-325756 | *safD* 325633-326103 |
| 4C | 210492-210495 | *stiA* 210160-210699 c |
| 5C | 210643-210647 | *stiA* 210160-210699 c |
| 4A | 210646-210467 | *stiA* 210160-210699 c |
| 4A | 210518-210522 | *stiA* 210160-210699 c |
| 5A | 210564-210568 | *stiA* 210160-210699 c |
| 4A | 210581-210584 | *stiA* 210160-210699 c |
| 5A | 210681-210685 | *stiA* 210160-210699 c |
| 5A | 209876-209880 | *stiB* 209429-210112 c |
| 5A | 210014-210018 | *stiB* 209429-210112 c |
| 4C | 209917-209920 | *stiB* 209429-210112 c |
| 4A | 209225-209228 | *stiC* 206856-209414 c |
| 4A | 209281-209284 | *stiC* 206856-209414 c |
| 4A | 209375-209378 | *stiC* 206856-209414 c |
| 4A | 209393-209396 | *stiC* 206856-209414 c |
| 4A | 206676-206679 | *stiH* 205768-206847 c |
| 6A | 206726-206731 | *stiH* 205768-206847 c |
| 4A | 206808-206811 | *stiH* 205768-206847 c |
| 4C | 206721-206724 | *stiH* 205768-206847 c |
| 5A | 358925-358929 | *stbE* 358286-359044 c |
| 4A | 359019-359022 | *stbE* 358286-359044 c |
| 4A | 360199-360202 | *stbD* 359010-360335 c |
| 4A | 360219-360222 | *stbD* 359010-360335 c |
| 5A | 360327-360331 | *stbD* 359010-360335 c |
| 4C | 360429-360432 | *stbD* 359010-360335 c |
| 5C | 362787-362791 | *stbC* 360340-362901 c |
| 4A | 362758-362761 | *stbC* 360340-362901 c |
| 4A | 362771-362774 | *stbC* 360340-362901 c |
| 4A | 362809-362812 | *stbC* 360340-362901 c |
| 6A | 363492-363497 | *stbB* 362885-363646 c |
| 4A | 363549-363552 | *stbB* 362885-363646 c |
| 5A | 363599-363603 | *stbB* 362885-363646 c |
| 4A | 363640-363643 | *stbB* 362885-363646 c |
| 4C | 364035-364038 | *stbA* 363708-364244 c |
| 4C | 364148-364151 | *stbA* 363708-364244 c |
| 4A | 364043-364046 | *stbA* 363708-364244 c |
| 6A | 364233-364238 | *stbA* 363708-364244 c |
| 4C | 584255-584258 | *fimI* 584229-584762 |
| 4A | 584263-584266 | *fimI* 584229-584762 |
| 4A | 584820-584823 | *fimC* 584806-584762 |
| 4A | 584993-584996 | *fimC* 584806-584762 |
| 6A | 585014-585019 | *fimC* 584806-584762 |
| 7C | 584877-584883 | *fimC* 584806-584762 |
| 5A | 585658-585662 | *fimD* 585529-588141 |
| 5A | 585704-585708 | *fimD* 585529-588141 |
| 5A | 588198-588202 | *fimH* 588156-589163 |
| 4A | 588261-588264 | *fimH* 588156-589163 |
| 4A | 588280-588283 | *fimH* 588156-589163 |
| 5A | 588328-588332 | *fimH* 588156-589163 |
| 4C | 588185-588188 | *fimH* 588156-589163 |
| 4A | 589189-589192 | *fimF* 589173-589691 |
| 4A | 589210-589213 | *fimF* 589173-589691 |
| 4A | 589286-589289 | *fimF* 589173-589691 |
| 4A | 589249-589352 | *fimF* 589173-589691 |
| 4A | 590267-590270 | *fimZ* 589737-590369 c |
| 5A | 590293-590297 | *fimZ* 589737-590369 c |
| 4A | 590305-590308 | *fimZ* 589737-590369 c |
| 4A | 591477-591480 | *fimY* 590973-591695 c |
| 4A | 591485-591488 | *fimY* 590973-591695 c |
| 4A | 592690-592693 | *fimW* 592187-592783 c |
| 4A | 592729-592732 | *fimW* 592187-592783 c |
| 4A | 592755-592758 | *fimW* 592187-592783 c |
| 5C | 2006388-2006392 | *csgC* 2006216-2006542 c |
| 5A | 2006366-2006370 | *csgC* 2006216-2006542 c |
| 4A | 2007045-2007048 | *csgA* 2006604-2007059 c |
| 4C | 2006980-2006983 | *csgA* 2006604-2007059 c |
| 4A | 2007362-2007365 | *csgB* 2007101-2007556 c |
| 4A | 2007461-2007464 | *csgB* 2007101-2007556 c |
| 5A | 2007552-2007556 | *csgB* 2007101-2007556 c |
| 4A | 2008424-2008427 | *csgD* 2008311-2008961 |
| 4A | 2008477-2008480 | *csgD* 2008311-2008961 |
| 6A | 2009422-2009427 | *csgF* 2009388-2009804 |
| 5A | 2009505-2009509 | *csgF* 2009388-2009804 |
| 4C | 2009489-2009492 | *csgF* 2009388-2009804 |
| 4C | 2009546-2009549 | *csgF* 2009388-2009804 |
| 4C | 2009886-2009889 | *csgG* 2009831-2010664 |
| 4C | 2009921-2009924 | *csgG* 2009831-2010664 |
| 4C | 2010287-2010290 | *csgG* 2009831-2010664 |
| 4A | 2009847-2009850 | *csgG* 2009831-2010664 |
| 4A | 2009859-2009862 | *csgG* 2009831-2010664 |
| 4A | 2010041-2010044 | *csgG* 2009831-2010664 |
| 5A | 2247332-2247336 | *stcD* 2246326-2247348 c |
| 4A | 2247342-2247345 | *stcD* 2246326-2247348 c |
| 4A | 2249732-2249735 | *stcC* 2247364-2249850 c |
| 5A | 2249818-2249822 | *stcC* 2247364-2249850 c |
| 4A | 2249843-2249846 | *stcC* 2247364-2249850 c |
| 5A | 2250365-2250369 | *stcB* 2249879-2250559 c |
| 6A | 2250458-2250463 | *stcB* 2249879-2250559 c |
| 5C | 2251070-2251074 | *stcA* 2250621-2251154 c |
| 4C | 2251097-2251100 | *stcA* 2250621-2251154 c |
| 5A | 2251061-2251065 | *stcA* 2250621-2251154 c |
| 4A | 2251092-2251095 | *stcA* 2250621-2251154 c |
| 5A | 2984449-2984453 | *steA* 2984435-2985022 |
| 5C | 2984559-2984563 | *steA* 2984435-2985022 |
| 4C | 2985172-2985175 | *steB* 2985102-2987801 |
| 4A | 2985120-2985123 | *steB* 2985102-2987801 |
| 6A | 2985227-2985232 | *steB* 2985102-2987801 |
| 4A | 2985258-2985261 | *steB* 2985102-2987801 |
| 4A | 2985275-2985278 | *steB* 2985102-2987801 |
| 4A | 2985295-2985298 | *steB* 2985102-2987801 |
| 4A | 2985306-2985309 | *steB* 2985102-2987801 |
| 4A | 2987831-2987834 | *steC* 2987814-2988587 |
| 5A | 2987923-2987927 | *steC* 2987814-2988587 |
| 4A | 2987935-2987938 | *steC* 2987814-2988587 |
| 5A | 2987961-2987965 | *steC* 2987814-2988587 |
| 4A | 2988631-2988634 | *steD* 2988607-2989113 |
| 5A | 2988687-2988692 | *steD* 2988607-2989113 |
| 4A | 2989131-2989134 | *steE* 2989128-2989598 |
| 4A | 2989153-2989156 | *steE* 2989128-2989598 |
| 4A | 2989256-2989259 | *steE* 2989128-2989598 |
| 4A | 3076986-3076990 | *stdC* 3076396-3077139 c |
| 4A | 3077109-3077113 | *stdC* 3076396-3077139 c |
| 4A | 3077111-3077114 | *stdC* 3076396-3077139 c |
| 5A | 3077132-3077136 | *stdC* 3076396-3077139 c |
| 4C | 3079526-3079529 | *stdB* 3077180-3079663 c |
| 4A | 3079621-3079624 | *stdB* 3077180-3079663 c |
| 4A | 3079657-3079660 | *stdB* 3077180-3079663 c |
| 4A | 3080259-3080262 | *stdA* 3079783-3080490 c |
| 4A | 3080352-3080355 | *stdA* 3079783-3080490 c |
| 4A | 3080397-3080400 | *stdA* 3079783-3080490 c |
| 4A | 3080414-3080417 | *stdA* 3079783-3080490 c |
| 4A | 3080446-3080449 | *stdA* 3079783-3080490 c |
| 5C | 3080227-3080232 | *stdA* 3079783-3080490 c |
| 5A | 3703808-3703812 | *lpfE* 3703495-3704022 c |
| 4A | 3703834-3703837 | *lpfE* 3703495-3704022 c |
| 4A | 3703886-3703889 | *lpfE* 3703495-3704022 c |
| 4A | 3704969-3704972 | *lpfD* 3704028-3705107 c |
| 4A | 3705024-3705027 | *lpfD* 3704028-3705107 c |
| 4A | 3705053-3705056 | *lpfD* 3704028-3705107 c |
| 5A | 3705097-3705102 | *lpfD* 3704028-3705107 c |
| 4C | 3705048-5705051 | *lpfD* 3704028-3705107 c |
| 4C | 3707567-3707570 | *lpfC* 3705125-3707653 c |
| 5A | 3708169-3708173 | *lpfB* 3707676-3708374 c |
| 4A | 3708227-3708230 | *lpfB* 3707676-3708374 c |
| 4A | 3708309-3708312 | *lpfB* 3707676-3708374 c |
| 4A | 3708316-3708319 | *lpfB* 3707676-3708374 c |
| 5A | 3708811-3708815 | *lpfA* 3708459-3708995 c |
| 5A | 3708966-3708970 | *lpfA* 3708459-3708995 c |
| 5A | 3708976-3708980 | *lpfA* 3708459-3708995 c |
| 5A | 3708985-3708989 | *lpfA* 3708459-3708995 c |
| 4A | 4570876-4570880 | *sefB* 4570869-4571609 |
| 5A | 4570908-4570912 | *sefB* 4570869-4571609 |
| 7A | 4570914-4570920 | *sefB* 4570869-4571609 |
| 7A | 4570950-4570956 | *sefB* 4570869-4571609 |
| 5A | 4570978-4570982 | *sefB* 4570869-4571609 |
| 4A | 4571632-4571635 | *sefC* 4571626-4574070 |
| 4A | 4571654-4571657 | *sefC* 4571626-4574070 |
| 4A | 4571684-4571684 | *sefC* 4571626-4574070 |
| 4A | 4571748-4571751 | *sefC* 4571626-4574070 |
| 4A | 4571814-4571817 | *sefC* 4571626-4574070 |
| 4C | 4574231-4574234 | *sefD* 4574067-4574513 |
| 5A | 4574110-4574114 | *sefD* 4574067-4574513 |
| 6A | 4574129-4574134 | *sefD* 4574067-4574513 |
| 4A | 4574175-4574178 | *sefD* 4574067-4574513 |
| 6A | 4574191-4574196 | *sefD* 4574067-4574513 |
| 4A | 4574220-4574223 | *sefD* 4574067-4574513 |
| 8A | 4574255-4574261 | *sefD* 4574067-4574513 |
| 7A | 4575220-4575226 | *sefR* 4574550-4575362 c |
| 5A | 4575254-4575258 | *sefR* 4574550-4575362 c |
| 5A | 4575301-4575305 | *sefR* 4574550-4575362 c |
| 4A | 4575316-4575319 | *sefR* 4574550-4575362 c |
| 4A | 4575336-4575339 | *sefR* 4574550-4575362 c |
| 5A | 4575341-4575345 | *sefR* 4574550-4575362 c |
| 8A | 4575349-4575356 | *sefR* 4574550-4575362 c |
| 4C | 4676973-4676976 | *sthE* 4675991-4677076 c |
| 4C | 4677516-4677519 | *sthD* 4677117-4677674 c |
| 4A | 4680069-4680072 | *sthC* 4677692-4680229 c |
| 4A | 4680078-4680081 | *sthC* 4677692-4680229 c |
| 4A | 4680114-4680117 | *sthC* 4677692-4680229 c |
| 4A | 4680134-4680137 | *sthC* 4677692-4680229 c |
| 4A | 4680158-4680161 | *sthC* 4677692-4680229 c |
| 7C | 4680062-4680068 | *sthC* 4677692-4680229 c |
| 4C | 4680205-4680208 | *sthC* 4677692-4680229 c |
| 4C | 4680775-4680778 | *sthB* 4680275-4680958 c |
| 4A | 4680867-4680870 | *sthB* 4680275-4680958 c |
| 5A | 4681389-4681393 | *sthA* 4681029-4681574 c |
| 4A | 4681481-4681484 | *sthA* 4681029-4681574 c |
| 4A | 4681559-4684562 | *sthA* 4681029-4681574 c |
