# Supplementary figures and images for: Analysis of the role of 13 major fimbrial subunits in colonisation of the chicken intestines by Salmonella enterica serovar Enteritidis reveals a role for a novel locus
Source: BMC Microbiol. 2008 Dec 18;8:228. doi: 10.1186/1471-2180-8-228 (PMC2644700; doi:10.1186/1471-2180-8-228)

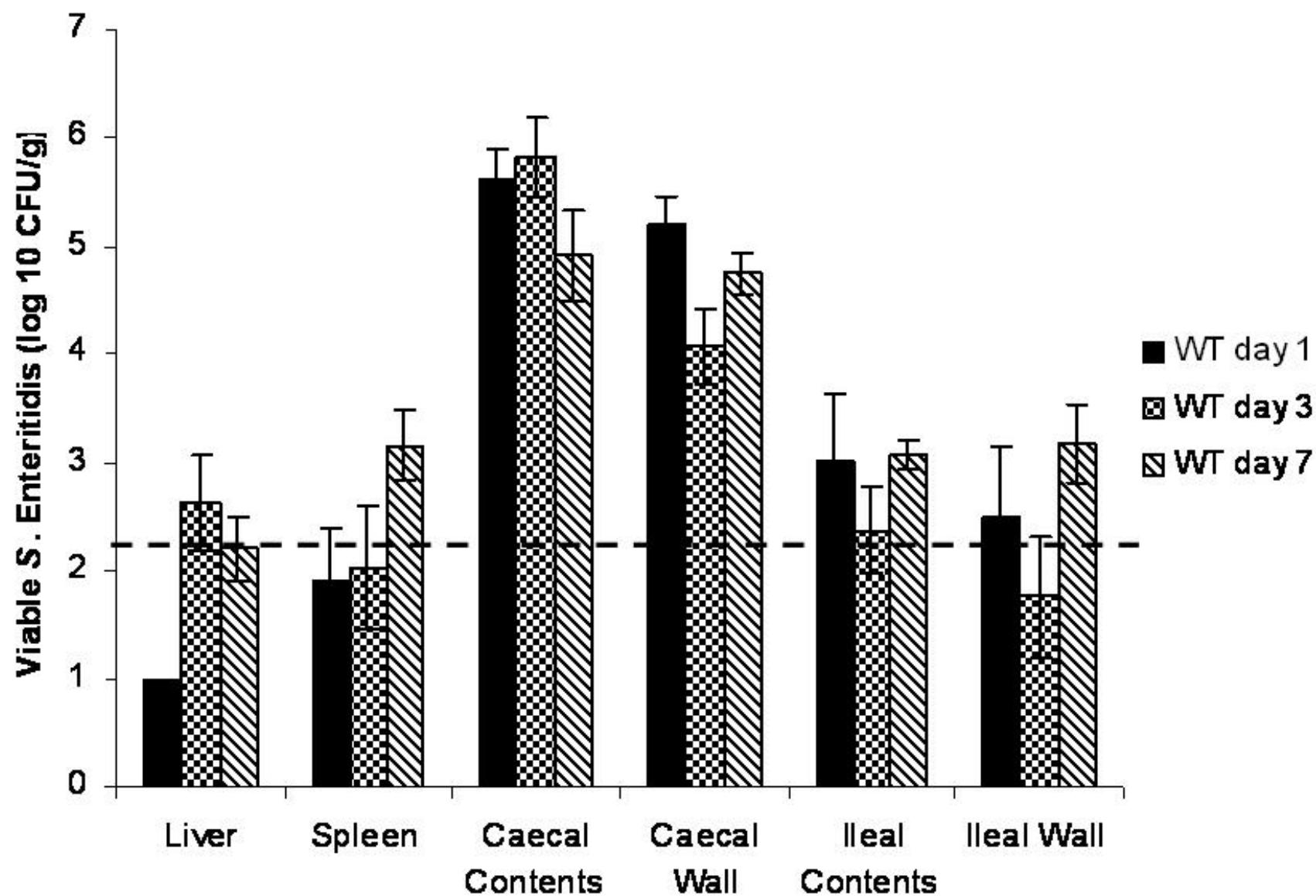

Supplement: Additional file 4 — S. Enteritidis P125109 colonisation of Rhode Island Red Chickens at 1, 3 and 7 days post-infection. The graph shows the colonisation of S. Enteritidis P125109 at different organs within the chicken, at different times post-infection. [file 1471-2180-8-228-S4.pdf]
